# Single-Molecule-Sensitive Three-Dimensional Atomic Heterostructures with Extreme Light-Matter Coupling

**DOI:** 10.1021/jacs.4c15029

**Published:** 2025-02-11

**Authors:** Yi-Jui Yeh, Shao-Yu Chen, Wesley Wei-Wen Hsiao, Yoshifumi Oshima, Mari Takahashi, Shinya Maenosono, Kuo-Lun Tung, Wei-Hung Chiang

**Affiliations:** † Department of Chemical Engineering, National Taiwan University of Science and Technology, Taipei 10607, Taiwan; ‡ Department of Chemical Engineering, National Taiwan University, Taipei 10607, Taiwan; § School of Materials Science, Japan Advanced Institute of Science and Technology, 1-1 Asahidai, Nomi, Ishikawa 923-1292, Japan; ∥ Sustainable Electrochemical Energy Development (SEED) Center, National Taiwan University of Science and Technology, Taipei City 10607, Taiwan

## Abstract

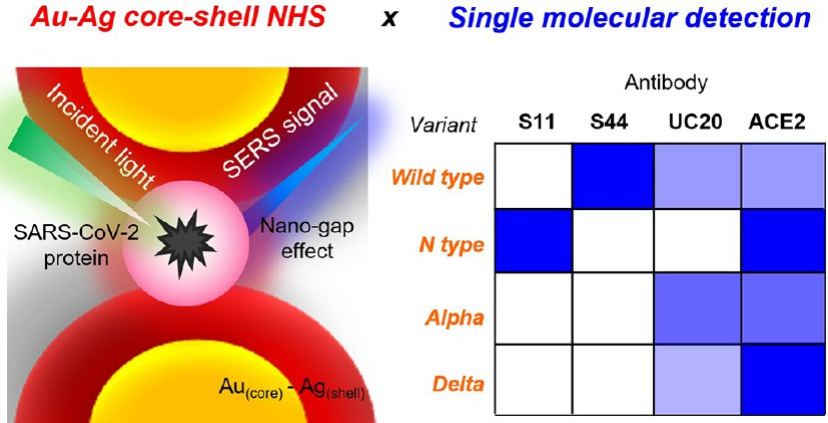

Three-dimensional heterostructures (3DHS) with controlled compositions and tuned properties are highly desired for fundamental studies and applications in optoelectronics, nanocatalysis, clean energy, and biomedicine. However, conventional nanostructure engineering is hindered by challenges such as poor structural control, time- and energy-intensive processes, the use of hazardous and expensive chemicals, and harsh conditions. Here, we report plasma-assisted epitaxy (PAE) engineering of a metal–organic 3DHS with extreme light-matter interaction for rapid single-molecule-level sensing. Plasmonic-active 3DHS composed of structure-tuned gold–silver core–shell nanoparticles (AuAgCSNPs) was precisely engineered using stable and scalable microplasma-enabled nanofabrication under ambient conditions. The engineered AuAgCSNP-based 3DHS possessed exceptional Raman enhancement under suitable laser excitation, leading to single-molecule detection of SARS-CoV-2 spike proteins in simulated human saliva via surface-enhanced Raman scattering (SERS). The developed plasma fabrication method allows the production of centimeter-scale SERS-active metal–organic 3DHS on disposable, flexible, lightweight, and cost-effective substrates, thereby opening a new avenue for next-generation biosensing, nanoelectronics, nanocatalysis, and biomedical applications.

## 1 Introduction

Rapid, sensitive, and cost-effective detection of drugs, antigens, and disease biomarkers is not only important for reducing the mortality of cancer and other diseases but also for promoting early medical diagnostics and discovery of new drugs.^1−4^ For example, the ongoing coronavirus disease 2019 (COVID-19) pandemic caused by the respiratory syndrome coronavirus 2 (SARS-CoV-2) has caused more than 775.5 million cases worldwide in May 2024.^5,6^ However, current detection methods, including enzymatic techniques, mass spectrometry , high-performance liquid chromatography, and electrochemical methods have been hampered by the problems of expensive chemicals, labor-intensive sample preparation, time- and energy-consuming steps, low throughput, low selectivity, and sensitivity.^7^ Nanostructure-enabled surface-enhanced Raman scattering (SERS) is a powerful light-matter coupling-enhanced approach for rapid and sensitive biosensing. In addition, controlling the light-matter interactions of SERS-active nanostructures can be a useful strategy for biomedical imaging and diagnostics, nanocatalysis, and clean energy conversion and generation.^1,8−10^ However, conventional preparations of SERS-active nanomaterials, including wet chemistry^1,11,12^ and vacuum-based dry methods such as electron beam lithography, focused ion beam patterning, and thermal evaporation,^1,9,13^ suffer from problems such as complicated and toxic chemicals, laborious procedures, uncontrolled stability and reproducibility of SERS sensor fabrication during multiple steps, and tedious and expensive equipment. Moreover, the SERS signal collection of those film-based SERS nanostructures is limited on the surfaces of substrates, making it challenging to improve the SERS sensitivity for biosensning.^14^

**Scheme 1:**
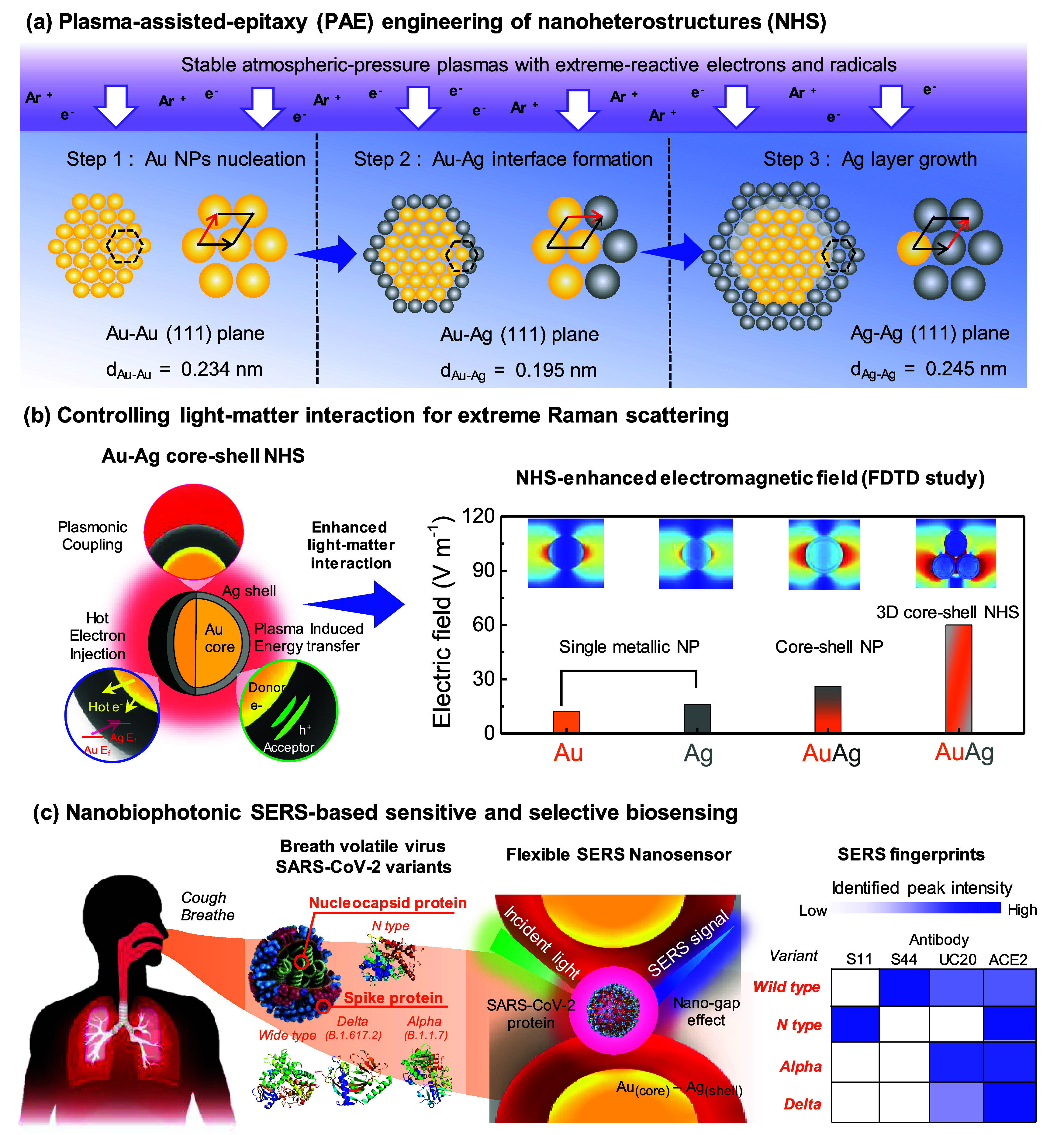
Plasma Engineering of Atomic AgAuCSNP-Based 3DHS with Tuned Light-matter Interactions for Rapid SERS-Based Single-Molecule Biosensing (a) Structure- and composition-controlled AgAuCSNPs with atomic-scale epitaxial heterostructures on cellulose papers were fabricated in a single step using solution-based PAE engineering under ambient conditions without the need for toxic chemicals, high-temperature and vacuum conditions. (b) Raman scattering can be extremely enhanced by tuning the light-matter interaction between the excitation laser and 3D Au–Ag core-shell NPs with controlled LSPR and large SERS-active volumes. (c) Au–Ag core-shell NPs can be used as nanobiophotonic biosensors and show exceptional performance for the SERS-based detections of spike proteins (S proteins) and nucleocapsid proteins (N proteins) SARS-CoV- 2 variants in simulated human saliva and cancer biomarkers.

Low-temperature plasmas, particularly microplasmas, are effective in nanomaterial synthesis and processing.^15−17^ Microplasmas represent a unique type of reactive nonthermal discharge characterized by the presence of energetic species, including electrons, ions, and radicals. This unique characteristic facilitates the precise engineering of nanostructures, including alloy nanoparticles, semiconductor quantum dots, and two-dimensional nanosheets with controlled compositions and structures on a minute scale.^18,19^ Herein, we report the plasma-assisted epitaxy (PAE) nanofabrication of metal–organic three-dimensional heterostructures (3DHS) with extreme light-matter coupling for sensitive and selective biosensing of SARS-VoC-2 variants and cancer biomarkers. Gold–silver core–shell nanoparticle (AuAgCSNP)-composed 3DHS was fabricated using a one-pot PAE engineering for 20 min under ambient conditions without the need for harsh chemicals, high temperature, or vacuum equipment (Scheme 1a). Outstanding SERS-active single-molecule detection of variant-specific SARS-CoV-2 proteins (including the nucleocapsid (N) protein and spike (S) protein) in simulated human saliva was achieved by optimizing the light-matter coupling, localized surface plasmon resonance (LSPR) electromagnetic fields, and porous SERS-active volumes of plasma-engineered 3DHS (Scheme 1b,c). Moreover, the developed technology allows fabrication of centimeter-scale SERS-active 3DHS on disposable, biodegradable, flexible, lightweight, cost-effectiveness, and porous substrates. This work provides an insight for atomic-controlled 3D heterostructures with tuned light-matter coupling for emerging applications including quantum optoelectronics, nanocatalysis, chemical and biosensing, cancer therapies and clean energy.^1,20−25^

## 2 Results and Discussion

**Figure 1:**
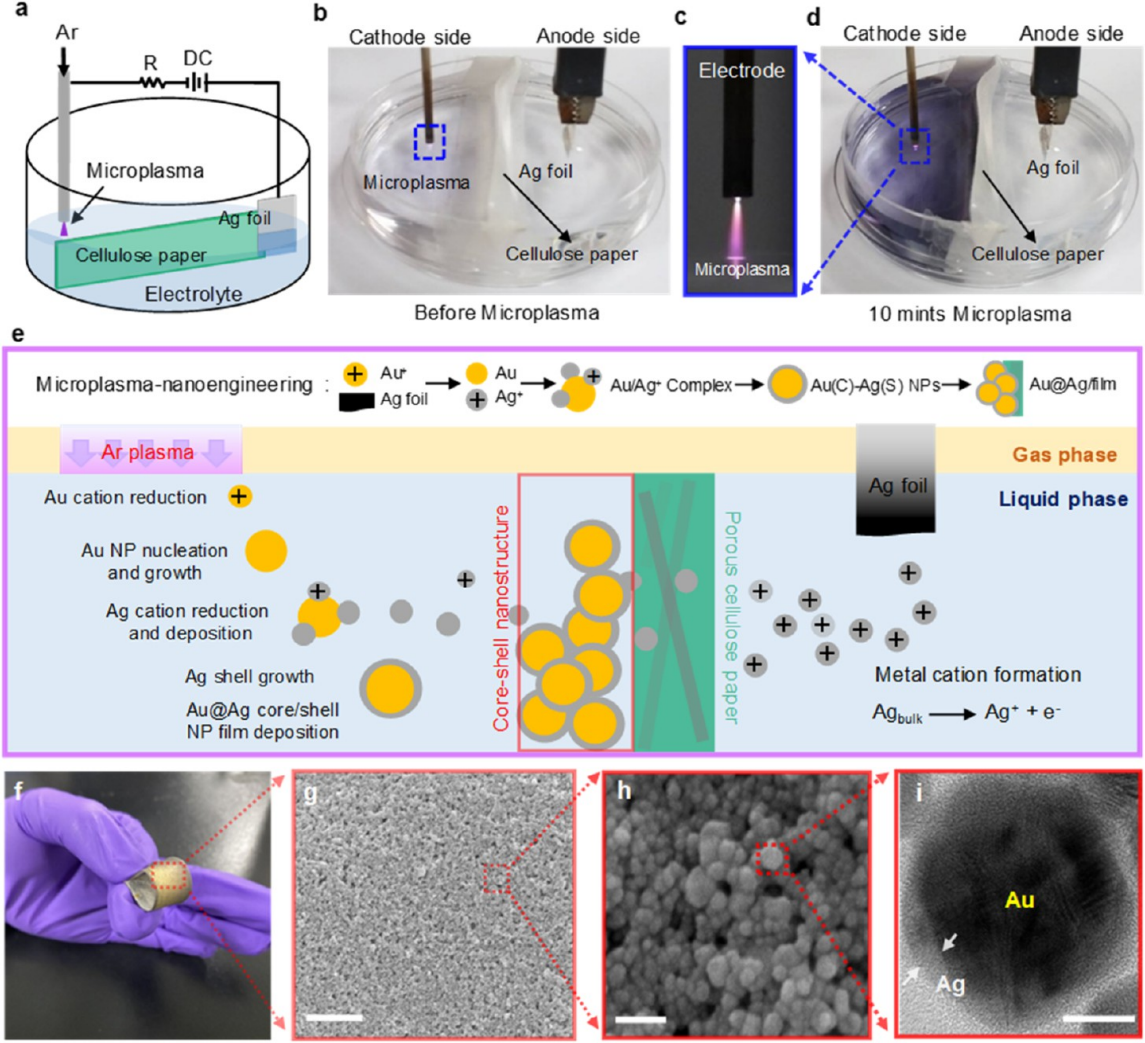
Plasma nanofabrication of flexible bimetallic Au–Ag core–shell NP-based 3DHS. (a) Illustration of the microplasma fabrication of Au–Ag core–shell NP-based 3DHS under ambient conditions. Photographs of a microplasma-liquid reactor for the Au–Ag core–shell NPs deposited on cellulose papers at (b) beginning and (d) 10 min. (c) Photograph of an Ar microplasma interacting with the liquid electrolyte. (e) Illustrations of the plasma synthesis of Au–Ag core–shell NP-based 3DHS. (f) Photograph of the as-fabricated flexible paper-based Au–Ag core–shell NP-based 3DHS. (g) Low-magnified and (h) high-magnified SEM images of top surfaces of the as-fabricated Au–Ag core–shell NP-based 3DHS. (Scale bar: 200 and 100 nm in (g) and (h), respectively. (i) Representative TEM image of one Au–Ag core–shell NP. Scale bar: 5 nm. Experimental parameters: current of 9 mA, a process time of 20 min, and 0.254 mM HAuCl_4_(_aq_))

Au–Ag core–shell NP-based 3DHS were fabricated using a direct current (DC) microplasma reactor. This reactor, which employed an Ar microplasma as the cathode, was operated under ambient environmental conditions. (Figure 1). In this setup, Ag foil served as the anode and Ag precursor, whereas the HAuCl_4_ aqueous solution acted as the electrolyte and Au precursor. Initially, the electrolyte exhibited transparency and colorless appearance (Figure 1b). Throughout this process, the plasma maintained its stability for effective interaction with the liquids (Figure 1c). After 10 min of operation, a discernible change occurred, with the electrolyte within the cathode cell acquiring a purple hue, whereas the anode cell remained colorless, indicating the formation of NPs induced by plasma treatment (Figure 1d). Notably, a transformation occurred on the surface of the paper aimed at plasma-treated batteries, displaying a metallic deep yellow hue, while the other side remained unaffected, indicating the presence of plasma-induced deposition on the cellulose paper (Figure 1e). The production rate of porous AuAgCSNP-based nanostructures on paper was estimated at 960 μg/(s·cm^2^) on cellulose papers following a single microplasma treatment of a 10 mL Au precursor solution (concentration: 7.5 mg/mL), with the potential for further enhancement through optimization of synthesis parameters such as plasma current, voltage, power, and treatment duration, or via the utilization of plasma arrays in conjunction with continuous-flow microfluidic devices. Following the synthesis, the treated papers underwent a 24-h drying period under ambient conditions while retaining their flexibility (Figure 1f). Subsequently, the SEM analysis (Figure 1g) revealed the formation of a paper-based porous metal NP-based network, characterized by dense and uniformly deposited particles. Further examination at higher magnification (Figure 1h) confirmed the presence of porous nanostructures within the deposited films, indicating successful one-pot preparation of the structure using microplasmas. Energy-dispersive X-ray spectroscopy (EDX) elemental mapping (Figure S1) of the as-fabricated sample demonstrated the uniform distributions of Au and Ag. Interestingly, Ag exhibited a broader distribution than Au, suggesting that the Au NPs were situated within the inner regions of the Ag particles. TEM analysis (Figures 1i and S2) provides additional evidence supporting the formation of Au–Ag core–shell NPs within the nanonetworks.

**Figure 2:**
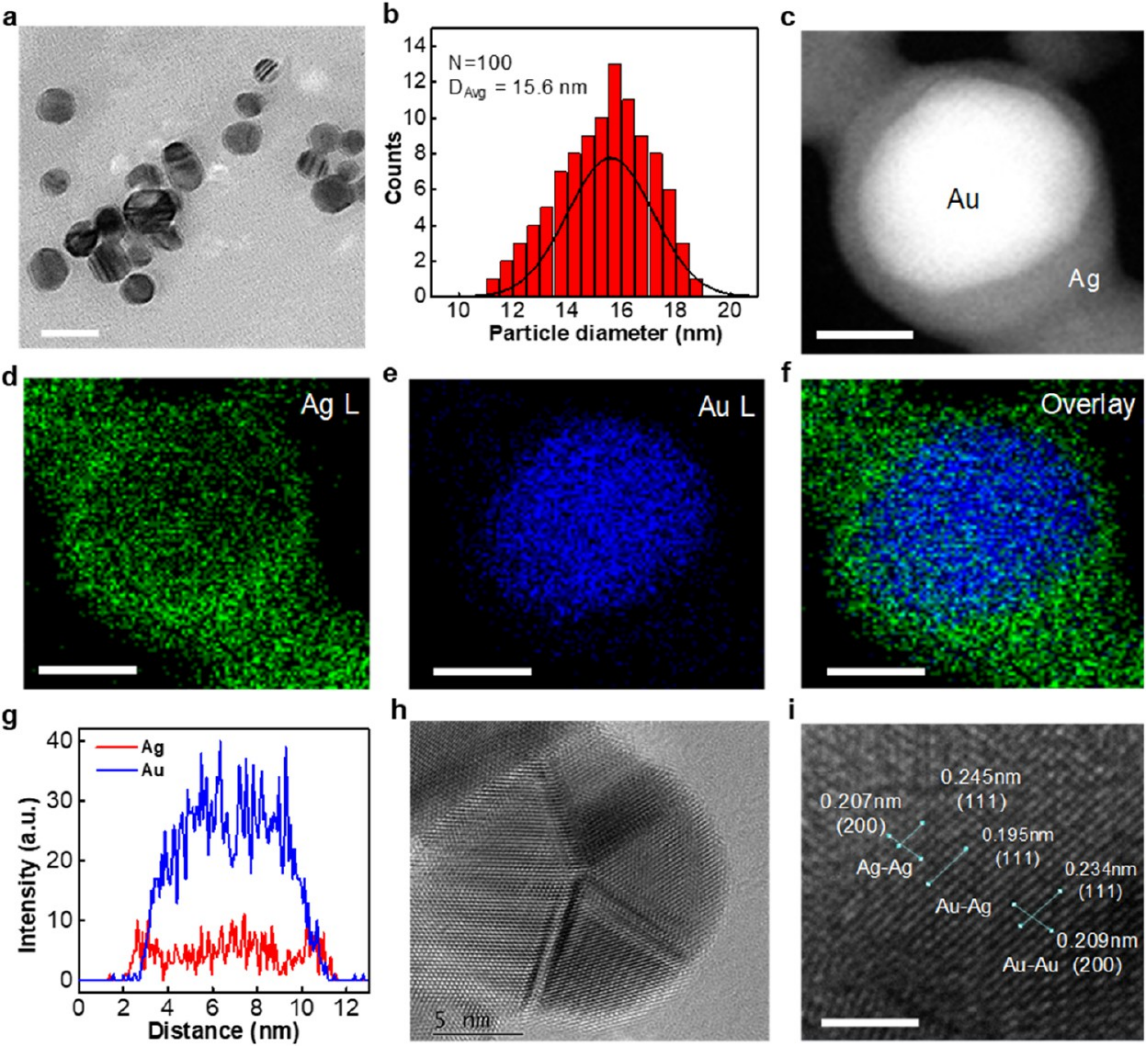
TEM characterization of Au–Ag core–shell NPs synthesized using microplasmas. (a) Representative low-magnification TEM image of the as-synthesized AuAgCSNP. Scale bar: 20 nm. (b) Histogram of particle-size distribution fitted with a Gaussian function. *D*_Avg_ indicates the averaged diameter of the particles. Histograms were analyzed for 100 NPs (*N* = 100). (c) Representative aberration-corrected STEM-HADDF image and STEM-EDX element maps of (d) Ag, (e) Au, and (f) overlay of as-synthesized AuAgCSNP-based 3DHS. Scale bar: 5 nm. (g) STEM-EDX line concentration profile for Au and Ag along a line across the as-fabricated AuAgCSNP. (h) Representative aberration-corrected HRTEM image of the Au NP. (i) Representative atomic-scale aberration-corrected HRTEM image of the interface region of the as-synthesized AuAgCSNP. Lattice fringes of the Ag–Ag, Ag–Au, and Au–Au phases are indicated. Scale bar: 1 nm. NP synthesis conditions were 9 mA plasma current, 20 min, and 0.254 mM HAuCl_4(aq)_.

AuAgCSNP-based 3DHS was prepared using the PAE method. Details of the preparation are provided in the Experimental Section and Supporting Information (See SI: Section S1–S2). SEM analysis (Figure 1g,1h) revealed the formation of a paper-based porous metal NP-based network characterized by dense and uniformly deposited particles. Transmission electron microscopy (TEM) analysis was conducted to investigate the morphological and crystalline characteristics of the synthesized AuAgCSNPs, as illustrated in Figure 2. Figure 2a presents a low-magnification TEM image, which illustrates the existence of spherical, particle-like nanostructures within the synthesized AuAgCSNPs (See SI: Figure S2). Histogram analysis (Figure 2b) indicates an average diameter of 15.6 nm with a standard deviation of 11.6%, showing a relatively narrow size distribution. Furthermore, Figure 2c presents a scanning TEM high-angle annular dark-field (STEM-HAADF) image of the AuAgCSNP. The image distinctly exhibits a brighter core of Au surrounded by a darker Ag shell, as the heavier Au atoms (atomic number, *Z* = 79) generate a brighter contrast than the lighter Ag atoms (*Z* = 47) in the dark-field imaging mode. Additionally, Figure 2d–f present the STEM-EDX elemental maps of Ag, Au, and their overlay, respectively, highlighting the presence of an Au core and Ag shell rather than the homogeneous distribution of Au and Ag throughout the entire nanoparticle.

To further characterize the elemental composition across individual AuAgCSNP, line-scan STEM-EDX analysis was employed to examine the edge, middle, and central regions along the cross section of a single nanoparticle. Figure 2g depicts the profiles of Au and Ag at different positions within the particle, indicating that the edge and central portions of the NP primarily consist of Ag and Au, respectively. Aberration-corrected atomic-scale high-resolution TEM was employed to examine Ag deposition on the Au cores within the as-prepared AuAgCSNPs. The specific HRTEM parameters are elaborated in the Experimental section. In Figure 2h, an illustrative HRTEM image depicts the Au core in its synthesized state, exhibiting twinned polycrystalline phases.^26^ This is consistent with prior studies involving the synthesis of Au nanoparticles that were also coated with citrate ligands via wet chemistry methods.^26^Figure 2i further shows the atomic-scale HRTEM image of the Au core, Au–Ag interfacial, and Ag shell regions of the individual AuAgCSNP (highlighted area in Figures 1i and S2–S5). The image presents well-resolved lattice fringes of the Ag (111), Au (111), and Au–Ag (111) planes with lattice constants of 0.245, 0.209, and 0.195 nm, respectively. The smaller Ag–Au lattice fringes observed in the interfacial (between the core and shell) regions of the probed AuAgCSNP are consistent with previous works.^27^ It is energetically favorable to transform Ag–Ag and Au–Au bonds to Ag–Au bonds in Au–Ag heterogeneous system.^27,28^ X-ray diffraction (XRD), Fourier-transform infrared (FTIR) spectroscopy, X-ray photoelectron spectroscopy (XPS), and absorbance spectroscopy were systematically performed to study the plasma-engineered AuAgCSNP-based 3DHS (See **SI:**Section 3–6 and Figures S6–S14). Overall, the above material characterization indicates that the AuAgCSNP-based 3DHS was successfully synthesized by microplsamas in one pot (see Figures S2–S5).

**Figure 3:**
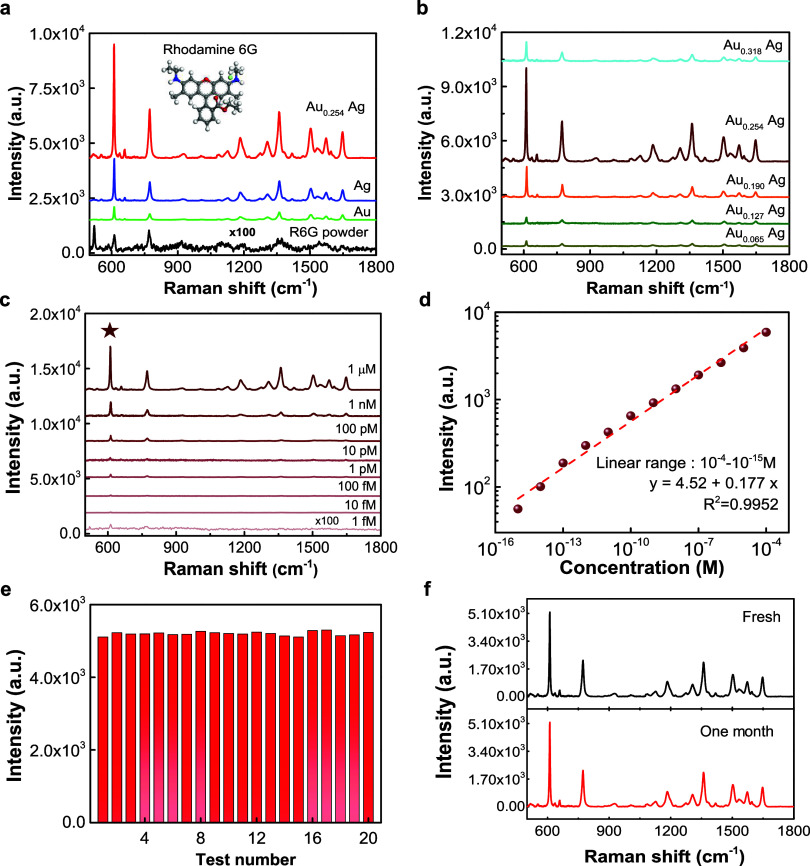
SERS study of the as-fabricated AuAgCSNP-based 3DHS. (a) Averaged SERS spectra of 10^–4^ M R6G with as-fabricated AuAgCSNP-based 3DHS, Ag, and Au. Raman spectrum of powder R6G is shown for comparison. (b) Averaged SERS spectra of 10^–4^ M R6G with different AuAgCSNP-based 3DHSs synthesized using various concentrations of HAuCl_4_. (c) Average SERS spectra of R6G at different concentrations (1 × 10^–9^–1 × 10^–15^ M) on the as-fabricated AuAgCSNP-based 3DHS. (d) Correlation between the average Raman intensity at 611 cm^–1^ and R6G concentrations ranging from 1 × 10^–4^ to 1 × 10^–15^ M. (e) Histogram of peak intensity at 611 cm^–1^ from SERS spectra of 10^–4^ M R6G acquired from 20 different paper samples within AuAgCSNP-based 3DHS. (f) Averaged SERS spectra of 10^–4^ M R6G collected from freshly prepared (top) and one-month storage (bottom) AuAgCSNP-based 3DHS.

To study the light-matter interaction of the as-synthesized AuAgCSNP-based 3DHS, micro-Raman spectroscopy with a 532 nm excitation wavelength was used (see SI: Figure S15). Rhodamine 6G (R6G) was used as the standard Raman probe due to its well-documented spectral characteristics under 532 nm excitation (See SI: Figure S16). In Figure 3a, the averaged Raman spectra acquired from an aqueous solution of R6G (10^–4^ M) on the fabricated AuAgCSNP-based 3DHS are depicted. Remarkably, the fabricated AuAgCSNP-based 3DHS demonstrated strong Raman enhancement across nearly all characteristic Raman peaks associated with R6G. The improvements observed are especially significant for the prominent peaks located at 611, 1360, 1510, and 1650 cm^–1^. These peaks correspond, in sequence, to the bending vibration of C–C–C rings and three distinct stretching modes of aromatic C–C bonds (1360, 1510, and 1650 cm^–1^).^29^ Comparative analysis revealed that AuAgCSNP-based 3DHS exhibited superior Raman enhancement compared to substrates composed solely of Ag or Au (Figure 3b). The unique Au–Ag core–shell nanostructure showed outstanding SERS activity by promoting hot electron densities on the Ag surface and diminishing the charge transfer resistance between the Au and Ag phases under appropriate laser excitation^11^ (See TEM discussion). Figure 3b shows the as-fabricated AuAgCSNP synthesized at 0.254 mM with the highest SERS response among all the NP samples, which was further used to study the LoD, uniformity, and stability of SERS. A range of R6G concentrations was used to estimate the LoD. As shown in Figure 3c, discernible peaks are observed in the Raman spectra of R6G at concentrations as low as 1 pM. Notably, at the lowest tested concentration (1 fM R6G), only a peak at 611 cm^–1^ was discernible, suggesting the single-molecule detection of R6G (See SI: Section S6).^33−36^Figure 3d shows the relationship between the intensity of the Raman peaks and the concentration of the R6G solution. The detectable linear range of R6G spans from 10^–4^ to 10^–15^ M. Due to the inherent nonlinearity of SERS and the porous structure of the paper substrate, the enhancement factor (EF) was estimated by comparing the Raman scattering intensity of the R6G peak at 611 cm^–1^ on the SERS substrate with that on the unmodified paper surface.^34,37^ The EF was determined to be within the range of 10^15^ (See SI: Section S7), which is comparable to previous studies (see Table S1). Figure 3e shows the Raman intensities at 611 cm^–1^ obtained from 20 distinct locations and indicates a relative standard deviation (RSD) of approximately 3%, demonstrating a consistency of the AuAgCSNP-based 3DHS. Moreover, Figures 3f and S17 shows the Raman spectra of freshly prepared and one-month storage samples, indicating that the Raman spectrum of R6G on the storage AuAgCSNP-based 3DHS was almost identical to that of the fresh substrate. The high stability of the fabricated AuAgCSNP-based 3DHS under ambient conditions can be explained by improved electronic charge transfer from Au to Ag (See SI: Figure S15).^55−58^ Overall, the plasma-engineered AuAgCSNP-based 3DHS exhibited outstanding SERS enhancement and improved chemical stability.

**Figure 4:**
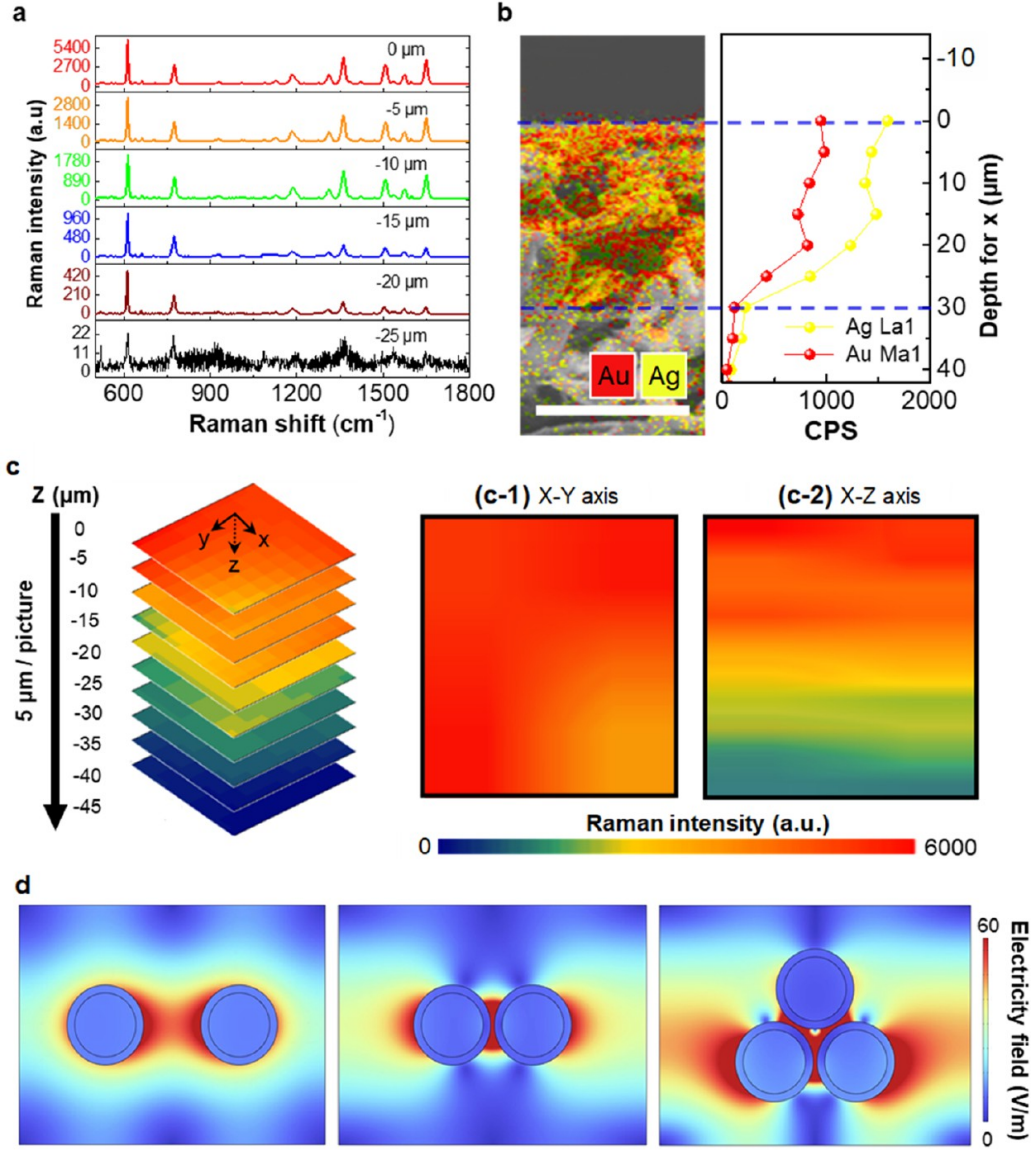
3D micro-Raman and FDTD studies of the AuAgCSNP-based 3DHS. (a) Average micro-Raman SERS spectra collected at different Z locations on the top surface of the fabricated AuAgCSNP-based 3DHS. (b) Cross-sectional view of SEM-EDX elemental map of the as-fabricated AuAgCSNP-based 3DHS. (Scale bar: 25 μm. Red: Au. Yellow: Ag.) The right panel shows the line profile analysis of the Au and Ag distribution across the selected region (indicated by dashed lines). (c) 3D confocal Z-stack micro-Raman maps obtained by examining the distribution of R6G molecules adsorbed on freshly prepared AuAgCSNP-based 3DHS. 2D micro-Raman maps of R6G adsorption on these substrates were generated, offering perspectives along both (c-1) the X-Y and (c-2) X-Z axes. A scale bar of 2 μm was used as the reference. Raman spectroscopy was conducted using a 10^–4^ M concentration of R6G as the analyte, employing a 532 nm laser for excitation, and integrating peak intensities at 611 cm^–1^ for mapping generation. (d) FDTD electric field distributions of Ag (left), Au (center), and AuAgCSNPs under 532 nm excitation. Furthermore, the study delves into the electric field distribution within the AuAg hybrid nanoparticles, analyzing variations across different interparticle distances, notably at (d) 15 and 1 nm. The figure also illustrates this. A configuration comprising three interconnected nanoparticles further contributes to our understanding of the electric-field dynamics in these nanostructures.^45^

We performed 3D micro-Raman tomography to study the spatial distribution of SERS hotspots in the fabricated AuAgCSNP-based 3DHS (See SI: Section S8). Figure 4a shows a comprehensive investigation of the SERS spectra that elucidates the differential absorption characteristics of R6G at variable depths (denoted as Z-positions) on meticulously engineered substrates. These substrates were characterized by the presence of AuAgCSNPs, which were strategically dispersed along the cellulose fibers. It was demonstrated that the intensity of the SERS signal decreased with an increase in substrate depth, transitioning from the proximal surface layer to a depth of 20 μm (*Z* = −20 μm). Notably, at a depth of 25 μm (*Z* = −25 μm), the R6G Raman signal becomes indiscernible. This phenomenon is indicative of a decrease in the concentration of plasmonic hotspots, which are integral to the SERS process and is attributed to the strategic deposition of AuAgCSNPs along the cellulose fibers of the paper-based substrate. The aforementioned hypothesis is further substantiated by SEM observations (Figure 4b), which delineate a gradational reduction in NP density from the substrate’s upper surface down to its midsection, thereby corroborating the observed trends in Raman signal intensity. Additionally, Figure 4c presents a compilation of 2D XY SERS mapping at various substrate depths, constructed through the accumulation of SERS signals corresponding to the 611 cm^–1^ Raman peak specific to R6G. The XY 2D Raman map revealed homogeneous Raman intensity across the substrate (Figures 4(c-1)). This uniformity is indicative of consistent adsorption of R6G molecules across the surfaces of the engineered substrates, suggesting uniform plasmonic characteristics. The X-Z map explored through 3D reconstruction (Figures 4(c-2)) reveals a high Raman intensity due to SERS effects from the substrate surface (*Z* = 0) to a depth of approximately 5 μm (*Z* = −5 μm), indicating a large-scale SERS zone exhibited in the fabricated substrates. This 3D micro-Raman analysis provided significant semiquantitative insights into the spatial distribution of hotspots in the produced SERS-active substrates, demonstrating their consistent, stable, and highly sensitive properties, attributable to the substantial active volume of SERS hotspots formed through the integration of AuAgCSNPs.

Finite difference time domain (FDTD) simulations were further utilized to study the electromagnetic interactions between the incident photons and an array of nanoparticles,^38−40^ including Ag, Au, and Ag–Au core–shell nanostructures, as shown in Figure S18. A comparison of the optical properties across these nanoparticles revealed a gradation in plasmonic effects, with a notable enhancement in the reflectance spectrum and distribution of localized electric fields, particularly around Ag, Au, and Ag–Au nanoparticles. Specifically, core–shell heterostructures exhibit superior electric field efficiency, underscoring the critical role of the localized surface plasmon effect in augmenting light-harvesting capabilities.^41,42^ This study provides a foundational understanding of the mechanisms by which plasmonic nanoparticles enhance the light-matter electromagnetic field, offering insights into the potential for further optimization of Raman scattering through nanostructure engineering.^43−46^ Detailed discussion is provided in the Supporting Information (See SI: Section S9 and Figure S18).

**Figure 5:**
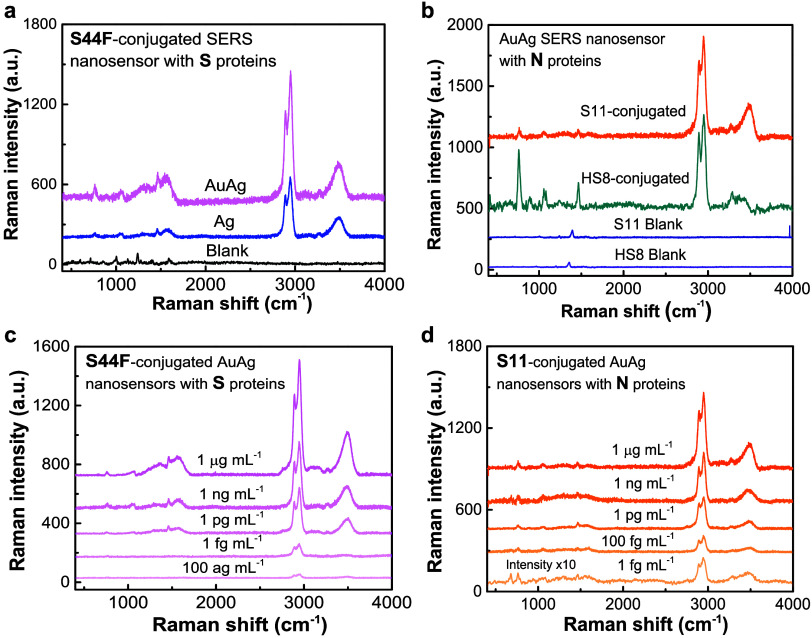
SERS detection of SARS-CoV-2 S and N proteins using antibody-functionalized AuAgCSNP-based 3DHS. (a) SERS spectra of SARS-CoV-2 S proteins with S44F-conjugated AuAgCSNP-based 3DHS and Ag NPs. (b) SERS spectra of SARS-CoV-2 N proteins with S11- and HS8-conjugated AuAgCSNP-based 3DHS. (c) SERS spectrum for SARS-CoV-2 S protein at different concentrations ranging from 1 μg mL^–1^ to 10 ag mL^–1^ on S44F-conjugated AuAgCSNP-based 3DHS. (d) SERS spectrum for SARS-CoV-2 N protein at different concentrations ranging from 1 μg mL^–1^ to 10 fg mL^–1^ on S11-conjugated AuAgCSNP-based 3DHS. (e) Raman intensity (a.u.) of different antibodies (S11, ACE, S44F, UC20) using the AuAg SERS nanosensor in the detection of variant-specific SARS-CoV-2 spike proteins. The bars represent the intensity levels corresponding to various antigens: N type, Wild type, Alpha variant, Delta variant, and a blank control.

To explore the potential applications of the synthesized AuAgCSNP-based 3DHS SERS sensors, a detailed investigation was undertaken, focusing on SARS-CoV-2 viral proteins (spike proteins (S proteins) and nucleocapsid proteins (N proteins)) in simulated human saliva (See SI: Section S10 and Figure S19). Figure 5 depicts the Raman spectra obtained from the interaction between the SARS-CoV-2 S protein (wild type) and S44F antibody-functionalized AuAgCSNP-based 3DHS (referred to as S44F-SERS). Upon conjugation of the S44F-SERS biosensors with the S protein of SARS-CoV-2, significant SERS signals were observed within the spectral profiles. These amplified signals were primarily associated with the vibrational stretching modes of the CH and CH_3_ groups within the spike protein, manifested at approximately 2895 and 2946 cm^–1^, respectively. The marked intensification of the SERS signal at the 2946 cm^–1^ peak is attributed to the robust adsorptive interactions between the AuAgCSNPs and CH_3_-containing functional groups, in conjunction with the electromagnetic field enhancement engendered by these groups upon excitation with a 532 nm laser. This observation is congruent with prior studies, reinforcing the utility of the developed Au–Ag SERS biosensors for the detection of the SARS-CoV-2 S protein (Figure 5a). The sensing efficiency was evaluated by quantifying the Raman peak intensity at 2946 cm^–1^ over the spectrum of SARS-CoV-2 S protein concentrations. A progressive increase in the Raman intensity was noted as the concentration of the target antigen varied from 100 ag mL^–1^ to 1.0 μg mL^–1^, as depicted in Figure 5c. Notably, a strong linear relationship was observed between the SERS intensity at 2946 cm^–1^ and the logarithm of the concentration of the target spike protein, as illustrated in Figure S20. The linear calibration curve was succinctly represented by the equation *y* = 75.85*x* + 990 log(C), with an *R*^2^ value of 0.9917. As elaborated in the Supporting Information, the LOD for the wild-type SARS-CoV-2 S protein was determined to be 100.0 ag mL^–1^. Furthermore, this study encompasses the utilization of SERS for the analysis of the nucleocapsid (N) protein of SARS-CoV-2, employing both HS8 human and S11 avian anti-N Immunoglobulin G (IgG) antibodies (Figure 5b). The evaluation of N protein solutions across a spectrum of concentrations revealed discernible SERS signals at notably low concentrations, notably at 1.0 fg mL^–1^, as illustrated in Figure 5d. The correlation between the concentration of N protein and the intensity of Raman peaks on AuAgCSNP-based 3DHS SERS substrates was found to be significantly linear, as exemplified by the regression equation for avian anti-N IgG antibodies: *y* = 74.69 log(*C*) + 1055. This equation delineated a linear detection range spanning from 1.0 fg mL^–1^ to 1.0 μg mL^–1^, accompanied by an *R*^2^ of 0.9938 (Figure S21). This study highlights the efficacy of S11-SERS biosensors in detecting minuscule concentrations of N protein, quantifiable down to 1.0 fg mL^–1^, with a pivotal emphasis on the peak at 2946 cm^–1^. Significantly, antibody-conjugated AuAgCSNP-based 3DHS enabled single-molecule SERS detection of SARS-CoV-2 S proteins. A detailed discussion can be found in SI Sections S7 and S10 and Tables S2–S4.

**Figure 6:**
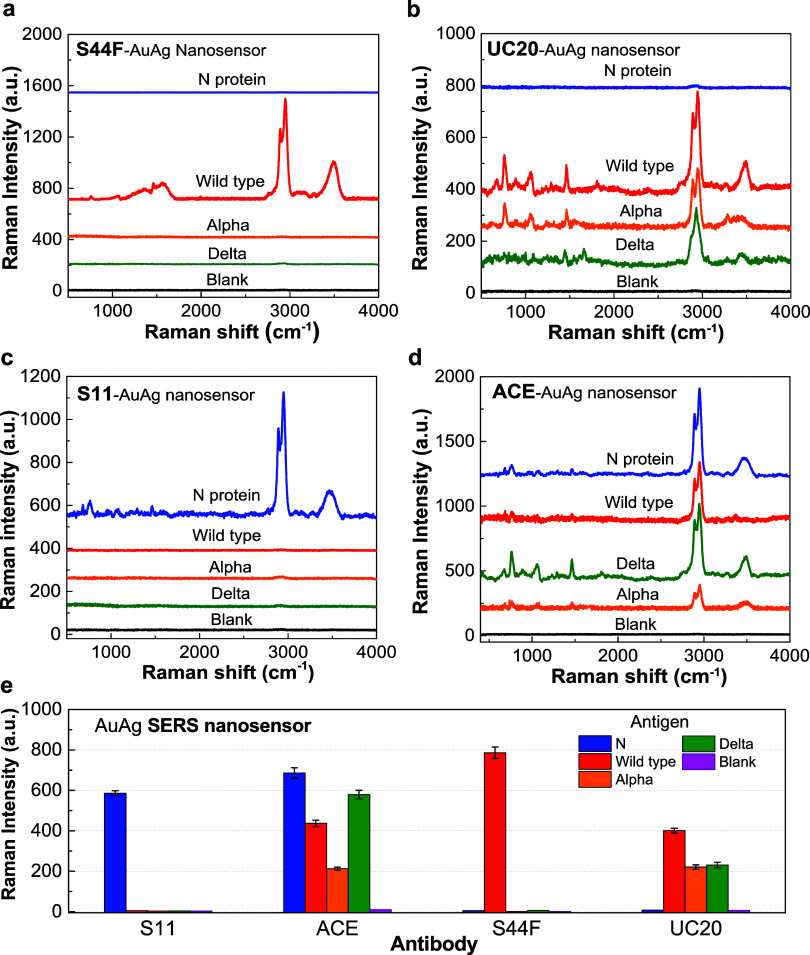
SERS responses elicited by antigen variants using antibody-functionalized AuAgCSNP-based 3DHS biosensors. The investigation entailed the employment of biosensors that were functionalized with a spectrum of antibodies, specifically (a) S44F, (b) ACE2, (c) UC20, and (d) N IgG (S11). (e) Raman intensity (a.u.) of different antibodies (S11, ACE, S44F, UC20) using the AuAg SERS nanosensor in the detection of variant-specific SARS-CoV-2 spike proteins. The bars represent the intensity levels corresponding to various antigens: N (Nucleocapsid), Wild type, α variant, Delta variant, and a blank control. This strategic conjugation was directed toward the identification of four distinct antigens: The N protein, along with the S proteins associated with the SARS-CoV-2 wild type, as well as its α and Delta variants. The experimental framework utilized artificial simulation of human saliva to serve as a medium for antigen detection. This analysis highlighted significant peak intensities at 2946 cm^–1^. To facilitate a uniform comparison across the various antigen–antibody interactions, the concentration of the target antigen within these assays was meticulously maintained at 1 μg mL^–1^.

The specificity of detection of different antibody-functionalized AuAgCSNP-based 3DHS SERS biosensors across diverse SARS-CoV-2 variants was studied, and the SERS responses are illustrated in Figure 6. Notably, SERS sensors functionalized with the S11 antibody demonstrated pronounced Raman scattering signals for the N protein, presumably due to the specific affinity between the S11 antibody and N protein, as depicted in Figure 6c. Conversely, SERS sensors functionalized with S44F exhibited marked Raman enhancement for the spike protein of the wild-type strain while displaying minimal Raman scattering for the N protein, as shown in Figure 6a. This pattern suggests pronounced specificity of the S44F antibody toward the wild-type spike protein, with minimal responses for the α and Delta variants. In contrast, ACE2-functionalized SERS sensors did not exhibit significant selectivity for either the N protein or variant spike proteins, as evidenced by the substantial Raman intensities for all tested N and S proteins, indicating potent interactions between ACE2 antibodies and the examined proteins (Figure 6d). Intriguingly, UC20-functionalized SERS sensors revealed enhanced Raman responses for the S protein across all examined variants, including the wild-type, Alpha, and Delta, thereby demonstrating effective specificity for the S variants (Figure 6b). These Raman spectroscopy findings are coherent and highlight the superior selectivity of our AuAgCSNP-based 3DHS SERS biosensors in identifying spike protein variants under simulated salivary conditions. Such discoveries are pivotal for the advancement of rapid and selective diagnostic approaches for SARS-CoV-2 variants, especially considering that conventional detection methods focused on the SARS-CoV-2 N protein may lead to false-positive results and inadvertently identify other coronaviruses, thereby underlining the significance of our research outcomes (see SI: Tables S3 and S4).^47,48^

**Figure 7:**
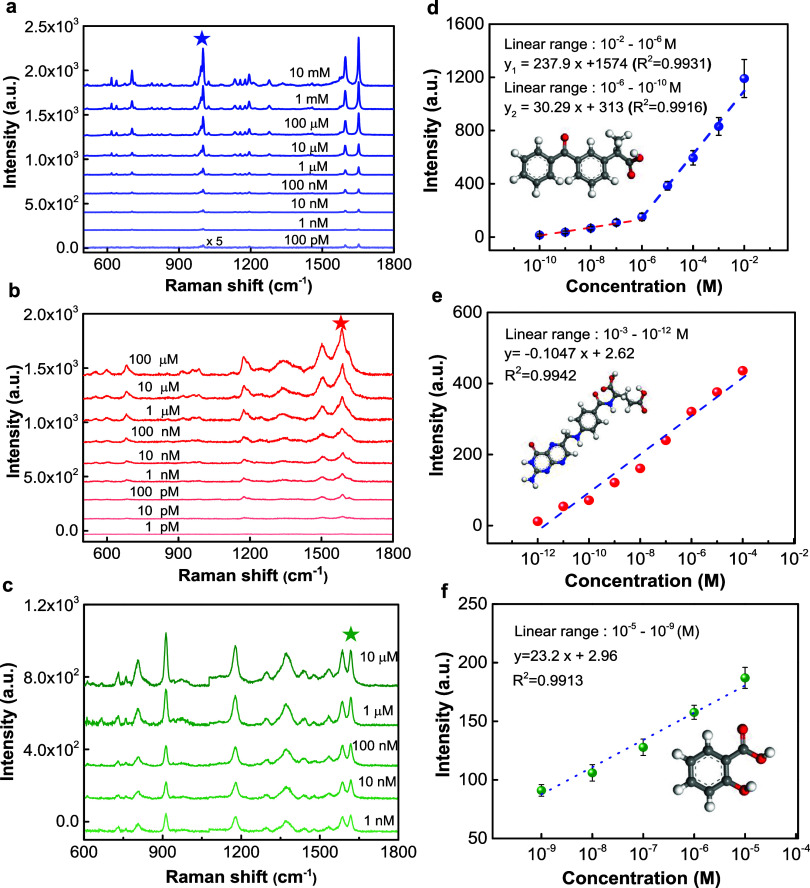
Label-free SERS detection of ketoprofen (KP), folic acid (FA), and salicylic acid (SA). Average SERS spectra of (a) KP, (b) FA, and (c) SA at different concentrations on the as-fabricated Au–Ag core–shell NP-based 3DHS. Correlations between averaged Raman intensities and (d) KP, (e) FA, and (f) SA at different concentrations. 532 nm laser was used for excitation.

Additionally, we studied the label-free detection of cancer biomarkers, including folic acid (FA), ketaprofen (KP), and salicylic acid (SA), using AuAgCSNP-based 3DHS (see SI**:** Section 11). FA is an essential vitamin in the human body, and its abnormal concentration can be linked to various cancers.^49,50^ KP is an effective nonsteroidal anti-inflammatory drug widely used in the treatment of inflammatory diseases. However, unsafe KP concentrations can induce heart attack, stroke, and stomach cancer. SA is an important biomarker for liver and kidney cancers.^51^ Rapid and sensitive detection of these biomarkers is particularly important for basic medical research and clinical applications. Figure 7 shows the SERS responses and sensing efficiencies of KP, FA, and SA facilitated by AuAgCSNP-based 3DHS. Specifically, Figure 7a–c demonstrate the SERS spectra for KP, FA, and SA with AuAgCSNP-based 3DHS. Each SERS spectrum shows a high signal-to-noise ratio over a spectrum of concentrations for KP, FA, and SA. Additionally, the examination revealed distinct linear correlations by associating the SERS intensities of the spectral bands of KA, FA, and SA (Figure 7d–f). Based on these results, the sensing ranges were 10^–2^ to 10^–10^ M, 10^–4^ to 10^–12^ M, 10^–2^ to 10^–9^ M for KP, FA, and SA, respectively. The SERS detection efficacy attained with the methodology demonstrated comparability with that of traditional detections and even surpassed that of other SERS-active materials in terms of sensitivity and reliability (see SI Tables S5–S10). The above SERS study indicates that AuAgCSNP-based 3DHS can be used for efficient and reliable SERS detection of large molecules, including viral proteins, as well as small molecules, including drugs and cancer markers. Moreover, the developed SERS detection provides a rapid detection rate of 450 runs per hour with a fixed laser excitation Raman measurement, showing the potential for a high-throughput sensing platform coupled with an automation system, compared with conventional methods.

## 3 Conclusions

3DHS with controlled properties and tuned light-matter coupling are important for optoelectronics, nanocatalysis, clean energy conversion and storage, and nanobiomedicine. Developing a scalable and controlled method that enables the precise atomic engineering of heterostructures is critical for fundamental studies and innovative applications. In this study, we report an effective plasma engineering method for fabricating 3DHS with extreme light-matter coupling for single-molecule detection. AuAgCSNP-composed 3DHS was fabricated using PAE engineering in a short time under ambient conditions without the need for harsh chemicals, high temperatures, and vacuum equipment. The engineered AuAgCSNP-based 3DHS showed extreme plasmonic light-matter coupling under 532 nm laser excitation, leading to rapid and sensitive single-molecule detection of variant-specific SARS-CoV-2 spike proteins in simulated human saliva using SERS. The epitaxial deposition of Ag on the Au cores leads to exceptional plasmonic properties. Moreover, the developed technology allows the fabrication of large-scale SERS-active metallic NP-based 3DHS on disposable, biodegradable, flexible, lightweight, cost-effective, and porous substrates using appropriate reactor design. This work not only provides a scalable and environmentally friendly method to fabricate flexible 3DHS but also offers a useful platform for the fundamental study of nanoparticle synthesis, nanostructure engineering for plasmonics, energy conversion and optoelectronics,^1^ and SERS-enabled sensitive diagnostics for personalized medicine.^24^

## 4 Experimental Section

### 4.1 Fabrication of AuAgCSNP-Based 3DHS

AuAgCSNP-based 3DHS was fabricated using a direct current (DC) microplasma reactor under ambient conditions. Figure S1 provides a schematic of the experimental setup. The reactor comprised a silver (Ag) foil anode submerged in an electrolytic solution alongside a microplasma cathode. Ag ions were dissolved from the Ag foil through plasma-induced electrochemical reactions. The distances between the anode and cathode and between the cathode and the liquid surface were approximately 3 cm and 2 mm, respectively. Cellulose paper served as a substrate for the deposition of NPs and was positioned between the two electrodes. The reactor was partitioned into two halves: a plasma-treating cell and an Ag-anode cell, separated by cellulose paper. A regulated flow of argon (Ar) gas, quantified at 25 standard cubic centimeters per minute (SCCM), was channeled through a stainless-steel capillary possessing a hollow structure, where the internal diameter was measured to be 180 μm (μm). This procedure was instrumental in the genesis and sustenance of microplasma. To catalyze and maintain a stable plasma entity, a high-voltage direct current (DC) power supply, specifically the UDC 5N30 (150 W) model from Hung Hui Technology, Taiwan, was judiciously coupled with a ballast resistor with 160 kilo-ohms (kΩ) resistance, ensuring a continuous flow of electric current pegged at 9 mA (mA). In this experimental setup, diverse concentrations of aqueous HAuCl_4_ solutions were utilized as the electrolytic medium and source of gold (Au) ions, with 0.1 molar (*M*) trisodium citrate (Na_3Ct) as a stabilizing agent. The operational time frame of this experiment was deliberately fixed at 20 min. (see SI: Section S2.)

### 4.2 Material Characterization

Absorbance spectra were acquired using a JASCO V676 absorbance spectrophotometer, employing a matched pair of quartz cuvettes with a path length of 1 cm. The respective solvents served as the baseline references for each spectral measurement. TEM analysis was conducted using a Philips Tecnai F20 G2 high-resolution transmission electron microscope operated at 200 kV. Prior to the TEM analysis, the prepared product solutions were diluted with deionized (DI) water and drop-cast onto lacey supported copper grids (Ted Pella, Inc., 300 mesh). Atomic resolution analytical TEM was performed using a JEOL JEM-ARM200F microscope equipped with a cold field emission gun and Cs corrector, operating in STEM mode with a resolution of 78 pm. EDX spectra were acquired from individual particles by focusing an electron beam on them. X-ray photoelectron spectroscopy (XPS) was conducted using a VG ESCA Scientific Theta Probe employing a pass energy of 50 eV, takeoff angle of 53°, and beam size of 400 μm. The samples were deposited on a silicon wafer via drop-casting and subsequently dried at 70 °C. High-resolution scanning electron microscopy (HR-SEM) was performed using a JEOL JSM-7900F scanning electron microscope equipped with an EDX detector. Fourier-transform infrared spectroscopy (FTIRzz) analysis was performed using a Nicolet FTIR-iS10 instrument equipped with an attenuated total reflection (ATR) module, covering a range of 500–4000 cm^–1^. The samples were directly deposited on the ATR stage for the measurement. X-ray diffraction (XRD) patterns were obtained using a Rigaku RINT-2500 diffractometer with Cu K radiation (λ = 1.5408 Å) operating at 40 kV/100 mA.

### 4.3 Micro-Raman Measurement

Micro-Raman scattering studies were carried out at ambient condition with a confocal micro-Raman spectrometer (JASCO NRS 5100) equipped with a 532 nm laser excitation. Before the measurement, the spectrometer was carefully calibrated using the silicon band at 520 cm^–1^ from a silicon wafer. The laser power was maintained at 0.45 mW throughout the experiment to avoid the thermal effect and the 5 s acquisition time. The spectra were background-subtracted and averaged from 100 random positions on each sample. This involved systematically scanning the XY plane of the samples and subsequently repeating the measurements at various Z-positions with a precision of 1 μm increment.^52^ The false-color XY maps were created through the scanning of a 532 nm laser beam across a 10 × 10 μm^2^ area, with a 1 μm step, while integrating Raman bands ranging from 609 to 613 cm^–1^. The details are provided in the Supporting Information. Our model assumes that the SERS signal primarily originates from molecules located within or very close to plasmonic hotspots, where the electromagnetic field is most intense.^53,54^

## Supporting Information Available

Experimental setup and additional analysis including XRD, FTIR, XPS, absorption spectroscopy, FDTD, TEM and Raman measurements (PDF)
