# Supplementary material for: Single-Molecule-Sensitive Three-Dimensional Atomic Heterostructures with Extreme Light-Matter Coupling
Source: J Am Chem Soc. 2025 Feb 11;147(10):8227–39. doi: 10.1021/jacs.4c15029 (PMC11912327; doi:10.1021/jacs.4c15029)
Supplement: Supplementary file 1 — ja4c15029_si_001.pdf [file ja4c15029_si_001.pdf]

Supporting Information

Single molecule-sensitive three-dimensional atomic heterostructures with extreme light-matter coupling

Yi-Jui Yeh[1,2], Shao-Yu Chen[2], Wesley Wei-Wen Hsiao[1], Yoshifumi Oshima[3], Mari Takahashi[3], Shinya Maenosono[3], Kuo-Lun Tung[2], and Wei-Hung Chiang[1,4*]

[1] Department of Chemical Engineering, National Taiwan University of Science and Technology, Taipei, 10607 Taiwan

[2] Department of Chemical Engineering, National Taiwan University, Taipei 10607, Taiwan

[3] School of Materials Science, Japan Advanced Institute of Science and Technology, 1-1 Asahidai, Nomi, Ishikawa, 923-1292 Japan

[4] Sustainable Electrochemical Energy Development (SEED) Center, National Taiwan University of Science and Technology, Taipei City 10607, Taiwan

*Corresponding author e-mail: whchiang@mail.ntust.edu.tw

(This supplementary information file contains 47 pages)

S1. Materials and chemicals.

Cellulose paper was purchased from Advantec International, Ltd. (Maidstone, UK). Tetrachloroauric acid ($HAuCl_4 \cdot 3H_2O$, 99.99% purity), sodium citrate tribasic dihydrate ($C_6H_5Na_3O_7 \cdot 3H_2O$, ≥99.0% purity), and silver foil (thickness 0.025 mm, 99.998% purity) were purchased from Alfa Aesar. Rhodamine 6G (99%) was purchased from Aldrich Co. All the chemicals were used directly without further purification. Bovine serum albumin (BSA) was obtained from Sigma Aldrich. Phosphate-buffered saline (PBS) buffer were prepared in-house. SARS-CoV-2 spike antibody (S44F), SARS-CoV-2 spike antibody (UC20), anti-human SARS-CoV-2 IgG nucleocapsid antibody (HS8), anti-chicken SARS-CoV-2 IgG nucleocapsid antibody (S11), human angiotensin-converting-enzyme 2 (ACE2), were purchased from Pharmtekx Co., Ltd, Taiwan. All reagents were used directly without further purification. All solutions were prepared with deionized (DI) water with a resistance of about 18 $k\Omega cm^{-1}$. Artificial saliva was prepared following the previous reports. Briefly, the solution of Na_2HPO_4 (0.6 g/L), anhydrous $CaCl_2$ (0.6 g/L), KCl (0.4 g/L), NaCl (0.4 g/L), urea (4 g/L), and mucin (4 g/L) were dissolved in deionized water and adjusted to pH 7.2, sterilized by autoclaving and stored at −4 °C until use.

S2. AuAgCSNP-based 3DHS Mechanism

In our study, the role of chloride ions in the electrolyte was carefully managed using a microplasma-assisted synthesis method, specifically designed to efficiently produce Au@Ag core-shell nanoparticles with low concentrations of metal ions. This method involves the rapid reduction of silver ions generated from silver foil in a plasma environment, which effectively minimizes the formation of silver chloride (AgCl). As depicted in **Figure 1**, the silver ions are immediately reduced upon formation, thereby significantly reducing the likelihood of AgCl precipitation. High-resolution transmission electron microscopy (HR-TEM) and energy-dispersive X-ray spectroscopy (EDS) mapping further confirm the formation of a well-defined Au@Ag core-shell structure, with only trace amounts of chlorine detected, uniformly distributed and not forming substantial AgCl precipitates. The presence of these trace amounts of chloride does not affect the surface-enhanced Raman scattering (SERS) performance, as evidenced by the strong and reproducible Raman signals obtained from the nanoparticles.

S3. XRD characterization

Fig. S6 shows the X-ray diffraction (XRD) pattern of the sample. The four distinct peak at 2 theta of 38.1°, 44.3°, 64.5°, and 77.3° could be assigned to the (111), (200), (220), and (311) crystalline planes of the AuAg bimetallic structures, respectively, while the other diffraction peaks at 34.2° and 46.8° were attributed to cellulose papers. Using the Scherrer equation ($d = K\lambda / \beta\cos\theta$)[1], the average particle sizes of Au-Ag NPs were estimated to be 10 and 21 nm for Au and Ag, respectively, which is consistent with the TEM observations.

S7. FTIR characterization

Fig. S7 presents additional FTIR spectra with 3391 and 2906 cm^{-1} of the stretching vibrations of the hydroxyl and C-H groups of cellulose, respectively,[2] suggesting that cellulose was preserved during the microplasma synthesis. The sample remained hydrophilic, providing a high absorption ability for probe molecules in aqueous solutions for Raman detection.

S8. XPS characterization

XPS was used to study the composition and survey spectra **(Fig. S8)** of the as-fabricated substrates show distinct peaks of Au, Ag, C, and O, which is in agreement with the TEM and XRD results (**Fig. S6**). High-resolution X-ray photoelectron spectroscopy (HRXPS) was performed to study the chemical states of the elements. The HRXPS spectrum of the Au 4f (**Fig. S9),** and Ag 3d regions (**Fig. S10)** show peaks of Au $4f_{7/2}$ (84.4 eV), Au $4f_{5/2}$ (88.4 eV), Ag $3d_{5/2}$ (368.4 eV), and Ag $3d_{3/2}$ (374.4 eV),[3, 4] indicating metallic states of Ag and Au were existed in the samples. Moreover, the C1s region of the blank paper shows a peak of sp^2 C=C (283 eV) and strong peaks of C-OH (285 eV) and C-O-C (287 eV) from the surface functional groups of cellulose, whereas the C1s region of the as-fabricated sample shows a strong peak of sp^2 C=C and a decreased peak of C-OH and C-O-C (**Fig. S11-S12),** suggesting that the surface functional groups of the cellulose fibers were reduced during the microplasma synthesis.

S6. Absorption spectroscopy study

In the microplasma synthesis, Au clusters were generated initially owing to the high reduction potential of Au, followed by Ag shell formation by Ag deposition on Au clusters. Because the Ag ions were solely from the Ag foil under the plasma conditions, the Ag ion concentration was fixed. In this study, the concentration of $HAuCl_4$ was varied to tune the size of the Au clusters. Absorbance spectroscopy was used to study the synthesis and optical properties of the as-synthesized samples. To clearly compare the optical properties of the samples, their absorbance spectra were normalized to the absorbance peak. **Fig. S13** shows the normalized absorbance spectra of the as-synthesized samples with varying $HAuCl_4$ concentrations in the electrolyte. The spectra exhibited 398 and 520 nm surface plasmon resonance (SPR) peaks for the as-synthesized Ag and Au NPs, respectively, which is consistent with previous work. After adding 0.318 mM $HAuCl_4$ to the electrolyte, the major SPR peak shifted to 414 nm and a small band appeared around 520 nm, suggesting that Ag atoms were incorporated with Au cores to form a hybrid nanostructure. The SPR peak was blue-shifted to 409 nm with a decrease in 0.254 mM $HAuCl_4$ concentration, and the band at around 520 nm was also reduced, suggesting that the Ag deposited on the Au surface gradually became thicker[5]. Notably, the two bands merged into a single SPR peak when the $HAuCl_4$ concentration was decreased to 0.190 mM. The appearance of a monomial SPR peak corresponding to Ag indicates that the Ag shell formed on the Au surface was homogeneous, and the optical contribution from the Au core was completely screened, which is consistent with a previous work[6]. Absorbance analysis confirmed that Au-Ag core-shell NPs could be synthesized using microplasmas.

To study the LSPR characteristics of the as-prepared core-shell NPs, precise control over the concentration of Ag ions was maintained under fixed plasma conditions, while the concentrations of Au ions were systematically varied by adjusting the concentration of HAuCl4. The synthesis process and the optical features of the resultant samples were investigated using absorbance spectroscopy. Absorbance analysis unequivocally demonstrated the successful synthesis of Au-Ag core-shell NPs with tailored LSPR properties, amenable to deposition onto paper substrates using microplasmas. According to the aforementioned results, it is envisaged that metal ions, radicals, and electrons from plasma will initially infiltrate the pores of the cellulose fiber papers. This infiltration is driven by electrostatic forces, leading to the attraction of metal ions to negatively charged surface functional groups on the cellulose fibers. Notably, Au ions undergo

reduction facilitated by plasma electrons, which is attributable to the lower reduction potential of Au compared to that of Ag. This reduction process succeeded by the nucleation of twinned Au clusters and subsequent particle growth aided by the presence of citrate as a stabilizing agent. Additionally, silver atoms are reduced from silver ions and subsequently deposited onto the surfaces of Au clusters through epitaxial growth mechanisms. (see TEM discussion), yielding a porous nanoassembly of Au-Ag core-shell NPs deposited on cellulose papers. The production rate of 3D Au-Ag core-shell NPs on paper was estimated to be 960 μg s^{-1} cm^{-2} using a single microplasma treatment of a 10 mL Au precursor solution (concentration: 7.5 mg/mL), with the potential for further enhancement through optimization of synthesis parameters such as plasma current, voltage, power, and treatment duration, or via the utilization of plasma arrays in conjunction with continuous-flow microfluidic devices.

S7. Enhanced factor calculation and single-molecule SERS detection

To estimate EF from the SERS-active substrates, the ratio of $(I_{SERS}/C_{SERS})/(I_R/C_R)$ was reported [7-9]. C_{SERS} is the concentration of the R6G solution on the SERS substrates, and C_R is the concentration of the R6G solution (10^{-4}M) that produces a spontaneous Raman signal on bare papers. In addition, I_{SERS} and I_R denote the integrated Raman intensities of R6G under the aforementioned experimental conditions. The calculation is as follows.

$$EF = (I_{SERS}/C_{SERS}) / (I_R/C_R).= (8.62/10^{-15}\,M)/(5.84/10^{-3}\,M) = \sim 1 \times 10^{15}.$$

To assess the feasibility of detecting a single molecule, calculations were conducted based on the SERS results. In a 10 mL solution of R6G at concentration of 10^{-15} M, approximately 6,022,000 molecules were present. The scanning area of the micro-Raman spectrum (a circle with a radius of 2 μm) was estimated at approximately 1.2566 x 10^{-11} square meters. Given a substrate area of 1 square centimeter (1 x 10^{-4} square meters), under these conditions, the number of R6G molecules potentially detectable by micro-Raman scanning was approximately ~0.76 molecules, which approaches a single molecule. [8, 10, 11] On the other hand, in a 25 mL solution of SARS-CoV-2 S proteins with a concentrations of ~100 ag/mL (molecular weight: 135 kDA) on a 0.01 square centimeter (1 x 10^{-6} square meters) of substrate area, there are approximately ~0.14 molecules of SARS-CoV-2 S protein in the Raman detecting area. In our work, the single-molecule-level detection of SARS-CoV-2 S protein was achieved using the as-fabricated AuAgCSNP-based 3DHS SERS sensors.

S8. 3D SERS study

To examine the spatial distribution of the SERS hotspots within the fabricated samples, we employed 3D micro-Raman tomography. This involved systematically scanning the XY plane of the samples and subsequently repeating the measurements at various Z-positions with a precision of 1 μm increment.[12, 13] The false-color XY maps were created through the scanning of a 532 nm laser beam across a 10 × 10 μm^2 area, with a 1 μm step, while integrating Raman bands ranging from 609 to 613 cm^{-1}.

S9. Finite Difference Time Domain (FDTD) study

Finite difference time domain (FDTD) simulations were performed to study the electromagnetic interactions between the incident photons and an array of nanoparticles, including Ag, Au, and Ag-Au core-shell nanostructures, as shown in **Figure S19**. These simulations provided insights into the optical absorbance and reflectance of the studied metal nanostructures under 532 nm excitation. A comparison of the optical properties across these nanoparticles revealed a gradation in plasmonic effects, with a notable enhancement in the reflectance spectrum and distribution of localized electric fields, particularly around Ag, Au, and Ag-Au nanoparticles (**Fig. S19(a-c)**). This enhancement suggests the potential of manipulating plasmonic interactions to achieve the desired optical near-field enhancement. Furthermore, the study and experimental results correspond, utilizing a simultaneous plasma process to fabricate nanoshells and structures, while also forming nanoporous materials. Consequently, the spatial distribution of the electric fields within the cellular structures containing coupled nanoparticles was examined. This examination demonstrated that SERS-active nanomaterials devoid of non-nanostructured nanoparticles, those containing coupled nanoparticles, and specifically those with three core-shell coupled nanoparticles derived from these nanoparticles exhibit a significantly enhanced electric field, as illustrated in **Fig.S19(d-f)**. This enhanced electric field, in turn, amplifies the absorption within the cellular matrix, directly contributing to an improvement in the efficiency of biosensors. This intensified field enhances absorption within the cellular matrix, directly contributing to the improved efficiency of the biosensors. Furthermore, this work applied the efficiencies of SERS-active nanomaterials incorporating nanostructured nanoparticles to those devoid of such nano-micro structures, highlighting a marked increase in efficiency with the introduction of metallic nanoparticles. Specifically, sensors integrated with core–shell structures exhibit superior electric field efficiency,

underscoring the critical role of the localized surface plasmon effect in augmenting light-harvesting capabilities. This study provides a foundational understanding of the mechanisms by which plasmonic nanoparticles enhance the performance of biosensors, offering insights into the potential for further optimization of sensor sensitivity through nanostructuring.

S10. SARS-CoV-2 SERS study

Anti-S IgG SARS-CoV-2 antibodies (S44F) (10 μL) were mixed into modified human saliva (pH = 7.4) at a concentration of 250 μg mL^{-1} and applied dropwise onto the SERS substrate. The functionalization reaction was conducted at a temperature of 4 °C over a period of 4 h. The substrate was then rinsed with PBS and DI water. Furthermore, nonspecific bindings were prevented by pipetting 5% BSA onto the resulting substrates, which were then incubated at 4 °C for 1 h. After successive rinsing with BSA and deionized water, the functionalized SERS substrates were stored at 4 °C prior to use. Human and chicken anti-N IgG SARS-CoV-2 antibodies (HS8 and S11, respectively), ACE2 and UC20 were functionalized on the SERS-active substrate using a same procedure. SARS-CoV-2 spike proteins were mixed into modified human saliva (pH 7.4) at various concentrations and then applied dropwise onto an anti-S IgG-conjugated SERS substrate. The immobilization reaction was conducted at a temperature of 4 °C over a period of 30 min. A similar procedure was used for the detection of SARS-CoV-2 N protein, in which various concentrations of N protein were applied dropwise on human or chicken anti-N IgG-functionalized SERS substrates. SERS spectra were then measured to evaluate the SERS response and calculate the LOD. SARS-CoV-2 N proteins and S proteins of different variants (wild-type, Alpha, and Delta) were mixed into stimulated human saliva solution at concentration of 1 μg mL^{-1}. The antigen solution was the applied dropwise onto different antibodies-functionalized SERS substrates. SERS spectra were then measured to evaluate the specificity and reproducibility of response towards antigen variants. SERS analysis was performed under ambient conditions using a confocal microRaman spectrometer (JASCO NRS 5100) at an excitation wavelength of 532 nm. Before obtaining measurements, the spectrometer was calibrated according to the silicon band at 520 cm^{-1}. Laser power was maintained at 0.45 mW throughout the 5-sec SERS experiment to avoid thermal effects. The spectra were obtained via background subtraction averaged using measurements from no fewer than 100 random positions on each sample.

Figure 5 depicts the Raman spectra obtained from the interaction between the SARS-CoV-2 S protein (wild-type) and S44F-functionalized gold-silver nanocomposites (referred to as S44F-SERS). Additionally, we performed Raman spectroscopic analysis of S44F-functionalized AuAg NPs for comparative purposes. Upon conjugation of the S44F-SERS biosensors with the S protein of SARS-CoV-2, significant SERS signals were discerned within the spectral profiles. These amplified signals were primarily associated with the vibrational stretching modes of the CH and CH_3 groups within the spike protein, manifested at approximately 2895 and 2946 cm^{-1}, respectively. The marked intensification of the SERS signal at the 2946 cm^{-1} peak is attributed to the robust adsorptive interactions between the AuAg nanostructures and CH_3-containing functional groups, in conjunction with the electromagnetic field enhancement engendered by these groups upon excitation with a 532 nm laser. This observation is congruent with prior studies, reinforcing the utility of the developed AuAg SERS biosensors for the detection of the SARS-CoV-2 S protein **(Fig. 5a)**. The biosensing was rigorously evaluated by quantifying the Raman peak intensity at 2946 cm^{-1} over a spectrum of SARS-CoV-2 S protein concentrations. A progressive increase in Raman intensity was noted as the concentration of the target antigen varied from 0.1 fg mL^{-1} to 1.0 µg mL^{-1}, as depicted in **Fig. 5c**. Notably, a strong linear relationship was observed between the SERS intensity at 2946 cm^{-1} and the logarithm of the concentration of the target spike protein, as illustrated in **Figure S21**. The linear calibration curve was succinctly represented by the equation y = 75.85x + 990 log(C), with an R^2 value of 0.9899. As elaborated in the supplementary materials, the LOD for the wild-type SARS-CoV-2 S protein in modified human saliva was determined to be 100.0 ag mL^{-1}. (**Table S7)**.

Furthermore, this study encompasses the utilization of SERS for the analysis of the nucleocapsid (N) protein of SARS-CoV-2, employing both human and avian anti-N immunoglobulin G (IgG) antibodies. Conjugation of the N protein with anti-N IgG antibodies on AuAg SERS substrates resulted in a pronounced SERS signal corresponding to the N protein, as evidenced by the Raman stretching vibrations of the CH and CN moieties within the N protein, manifesting at approximately 2895 and 2946 cm^{-1}, respectively. The evaluation of the N protein solutions across a spectrum of concentrations revealed discernible SERS signals at notably low concentrations, notably at 1.0 fg mL^{-1}, as illustrated in **Fig. 5d** and **Fig. S22**. The correlation between the concentration of N protein and the intensity of Raman peaks on AuAg SERS substrates was found to be significantly linear, as exemplified by the regression equation for avian anti-N IgG antibodies: y = 74.69 log(C) + 1055. This equation delineates a linear detection range spanning

from 1.0 fg mL^{-1} to 1.0 µg mL^{-1}, accompanied by R^2 of 0.9975. These observations are elaborated upon in the Supplementary Information section. This study highlights the efficacy of S11-SERS biosensors in detecting minuscule concentrations of N protein, quantifiable down to 1 fg mL^{-1}, with a pivotal emphasis on the peak at 2946 cm^{-1}. (See **SI: Tables 8 and 9**).

The specificity of the novel SERS biosensors was rigorously investigated through the strategic integration of variant-specific antibodies, enabling precise differentiation among various strains of the SARS-CoV-2 virus, including the original strain, alongside the Alpha and Delta variants. In this comprehensive study, AuAgCSNP-based 3DHS SERS sensors with distinct antibody functionalities, specifically S44F, ACE2, UC20, and S11, were employed to evaluate the proficiency of SERS immunoassays in the context of potential protein interference commonly present in human saliva. To emulate authentic physiological conditions, experimental assays were performed using synthetic saliva, with the intensity of the Raman band at 2946 cm^{-1} serving as the benchmark for assessing the detection capabilities of SERS biosensors when incubated with various antigens. The specificity detection corresponding to different antibody-functionalized SERS biosensors across diverse antigens is illustrated in **Fig. 6**. Notably, SERS sensors functionalized with the S11 antibody demonstrated pronounced Raman scattering signals for the N protein, presumably due to the specific affinity between the S11 antibody and the N protein, as depicted in **Fig. 6a** Conversely, SERS sensors functionalized with S44F exhibited marked Raman enhancement for the spike protein of the wild-type strain while displaying minimal Raman scattering for the N protein, as shown in **Fig. 6b**. Intriguingly, UC20-functionalized SERS sensors revealed enhanced Raman responses for the S protein across all examined variants, including the wild-type, Alpha, and Delta, thereby demonstrating effective specificity for the S variants **(Fig. 6c)**. This pattern suggests pronounced specificity of the S44F antibody towards the wild-type spike protein, with minimal responses for the Alpha and Delta variants. In contrast, ACE2-functionalized SERS sensors did not exhibit significant selectivity for either the N protein or variant spike proteins, as evidenced by the substantial Raman intensities for all tested N and S proteins, indicating potent interactions between ACE2 antibodies and the examined proteins **(Fig. 6d)**. These Raman spectroscopy findings are coherent and highlight the superior selectivity of our SERS biosensors for identifying spike protein variants under simulated salivary conditions. Such discoveries are pivotal for the advancement of rapid and selective diagnostic approaches for SARS-CoV-2 variants, especially considering that conventional detection methods focused on the SARS-CoV-2 N protein

may lead to false-positive results and inadvertently identify other coronaviruses, thereby underlining the significance of our research outcomes.[14-17].

S11. Label-free cancer biomarker SERS detection

To explore the utility of the synthesized SERS sensors for potential applications, a detailed investigation was undertaken focusing on a variety of cancer biomarkers, including KP, FA, SA, and SARS-CoV-2 proteins. **Figure 7** shows the SERS responses and sensing proficiencies of KP, FA, and SA facilitated by the utilization of nanosensors. Specifically, **Fig. 7a** through **Fig. 7c** shows the SERS spectra for KP, FA, and SA, utilizing AuAgCSNP-based 3DHS nanosensors. Each SERS spectrum shows a high signal-to-noise ratio over a spectrum of concentrations for KP, FA, and SA. Additionally, the examination revealed two distinct linear correlations by associating the SERS intensity of the spectral band, situated at approximately 1000 cm^{-1}, with the concentration of KP (C_{KP}), ascertained through linear regression analysis. The first linear fitting, as shown in **Fig. 7d**, can be expressed as $y = 1574 + 237.9 \log(C_{KP})$ (spanning from 10^{-2} to 10^{-6} M, R^2 value: 0.9931), while the second linear fitting is represented as $y = 313 + 30.3 \log(C_{KP})$ (ranging from 10^{-6} to 10^{-10} M, R^2 value: 0.9916). Similarly, for FA detection, the average Raman peak intensity at the strongest peak (1587.3 cm^{-1}) was plotted against the FA concentration (C_{FA}), yielding a linear fitting equation of $y = 2.62 - 0.147 \log(C_{FA})$ (covering concentrations from 10^{-4} to 10^{-12} M, R^2 value: 0.9942). Moreover, for SA detection, linear fitting was established with the equation $y = 1255 – 142.6 \log(C_{SA})$ (ranging from 10^{-2} to 10^{-9} M, R^2 value: 0.9923), as illustrated in **Fig. 7f**. Based on these linear fittings, the LoDs for KP, FA, and SA were estimated to 10^{-10}, 10^{-9}, and 10^{-9} M, respectively. Our investigation into SERS indicates that employing Au-Ag NP with a heterostructure as a functionalized layer on a paper substrate presents an efficient and reliable SERS modality for the sensitive detection of compounds such as KP, FA, and SA. Significantly, the SERS detection efficacy attained with this methodology is comparable to that of traditional detection approaches, and surpasses that of other SERS-active materials in terms of sensitivity and reliability. **(**see SI: **Table S2-S6)**.

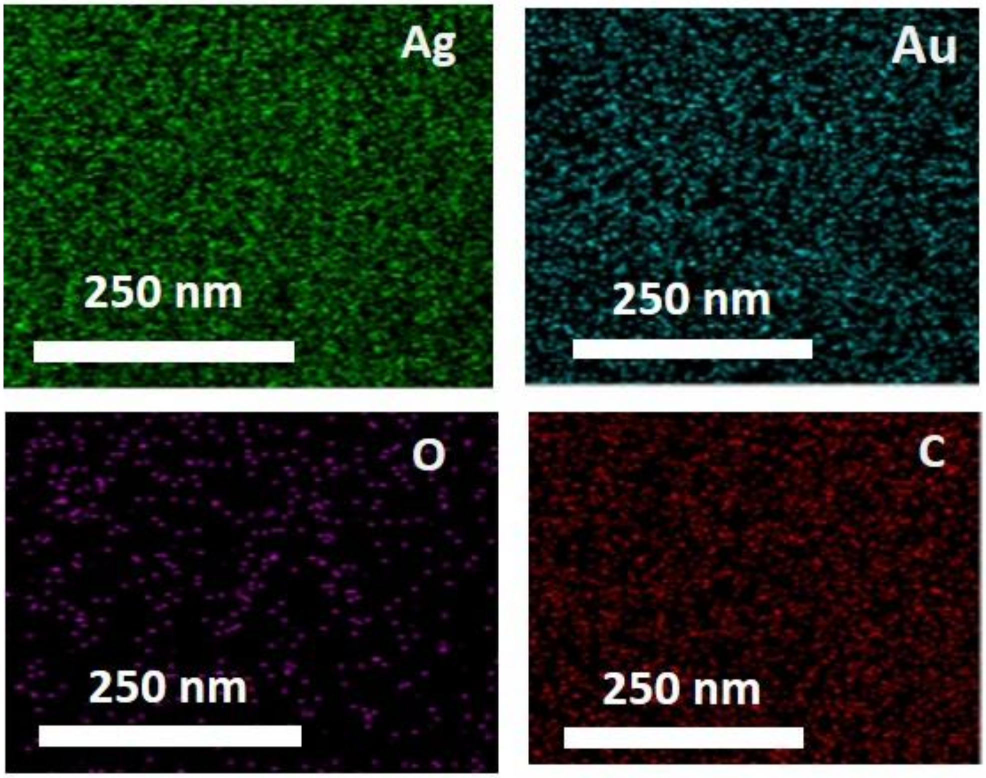

Figure. S1 SEM-EDS element maps of Ag, Au, O, and C for Au-Ag core-shell NPs on cellulose paper. Experimental conditions: 9 mA plasma current, 20 min process time, and 0.254 mM aqueous $HAuCl_4(_{aq})$.

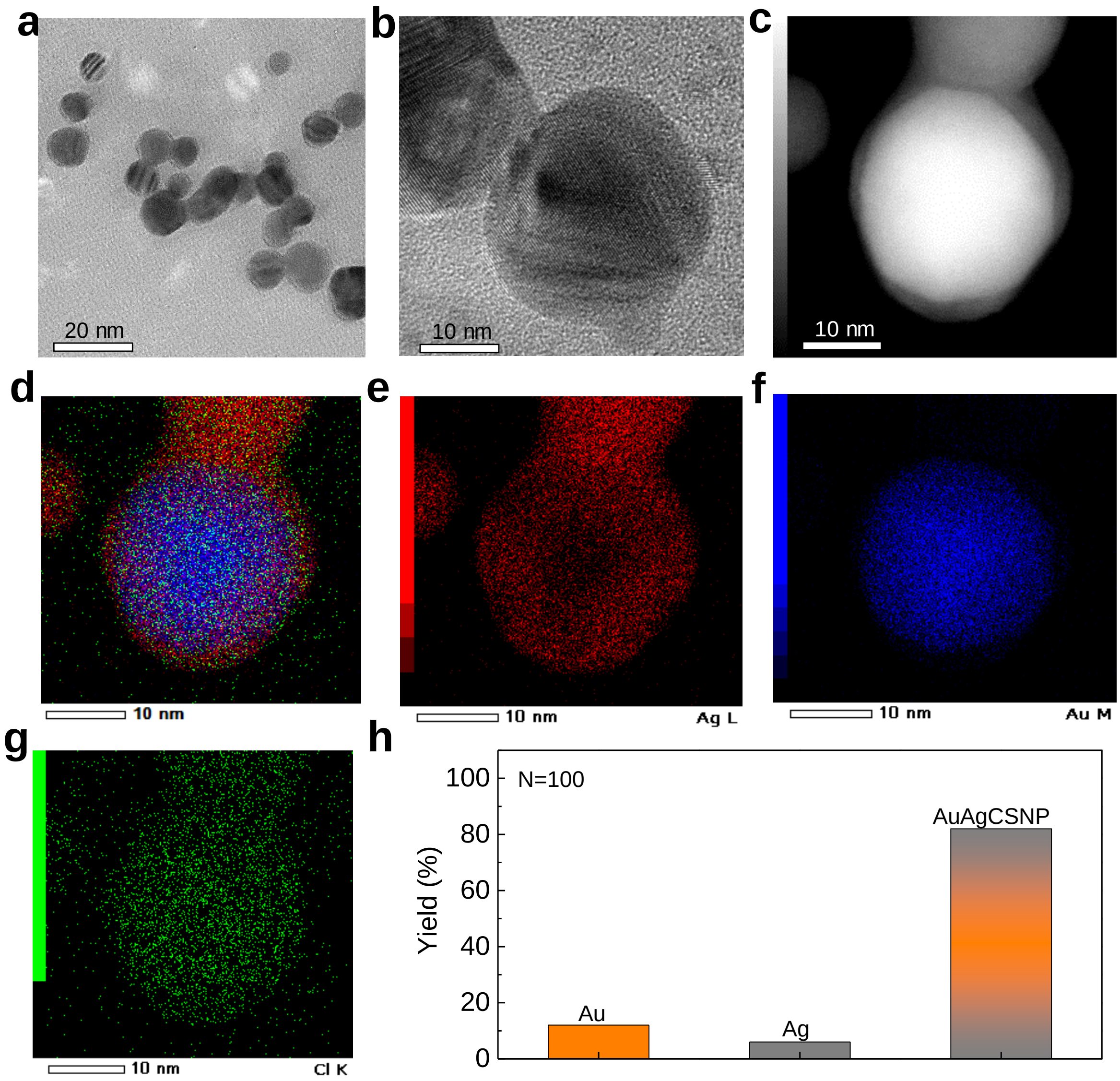

Figure S2. TEM structural and elemental analysis of AuAgCSNP. (a) Representative TEM image of plasma-synthesized AuAgCSNPs. (b) HRTEM image of AuAgCSNP. (c) Representative HAADF TEM images of a representative AuAgCSNP. (d) Overlay STEM EDX elemental map of Ag (red), Au (blue), and Cl (green). (e-g) Individual STEM EDX elemental maps showing the distribution of (e) Ag (L-edge, red), (f) Au (M-edge, blue), and (g) Cl (K-edge, green) within the nanoparticle showing in (d), confirming the formation of the Au core, Ag shell, and the presence of Cl in the structure. (h) Yield percentage of different nanoparticle structures (Au, Ag, and AuAgCSNP) synthesized using the plasma method. The data is based on 100 particles observed using TEM.

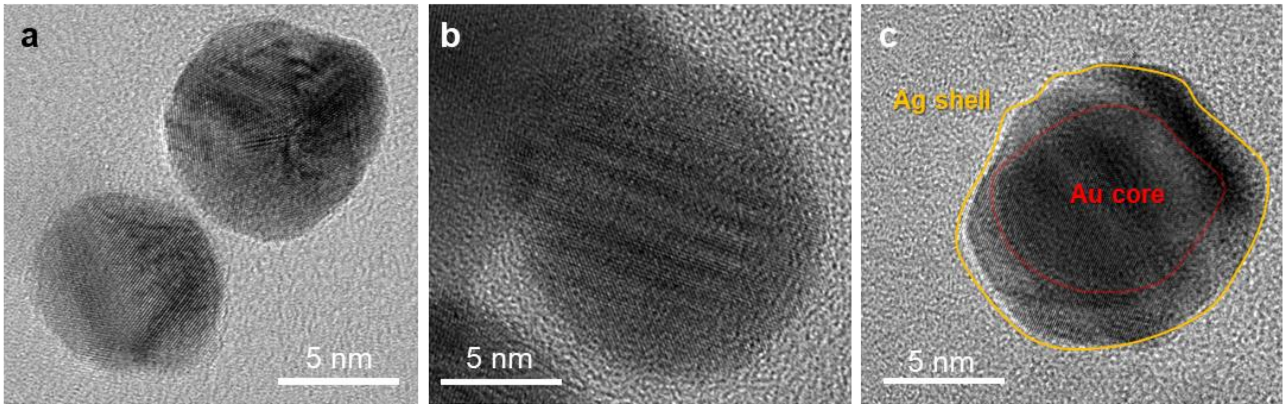

Figure S3. Representative TEM images of (a) Ag NPs, (b) Au NPs, and (c) AuAgCSNP. The Au core and Ag shell are labeled to indicate the location in the particle.

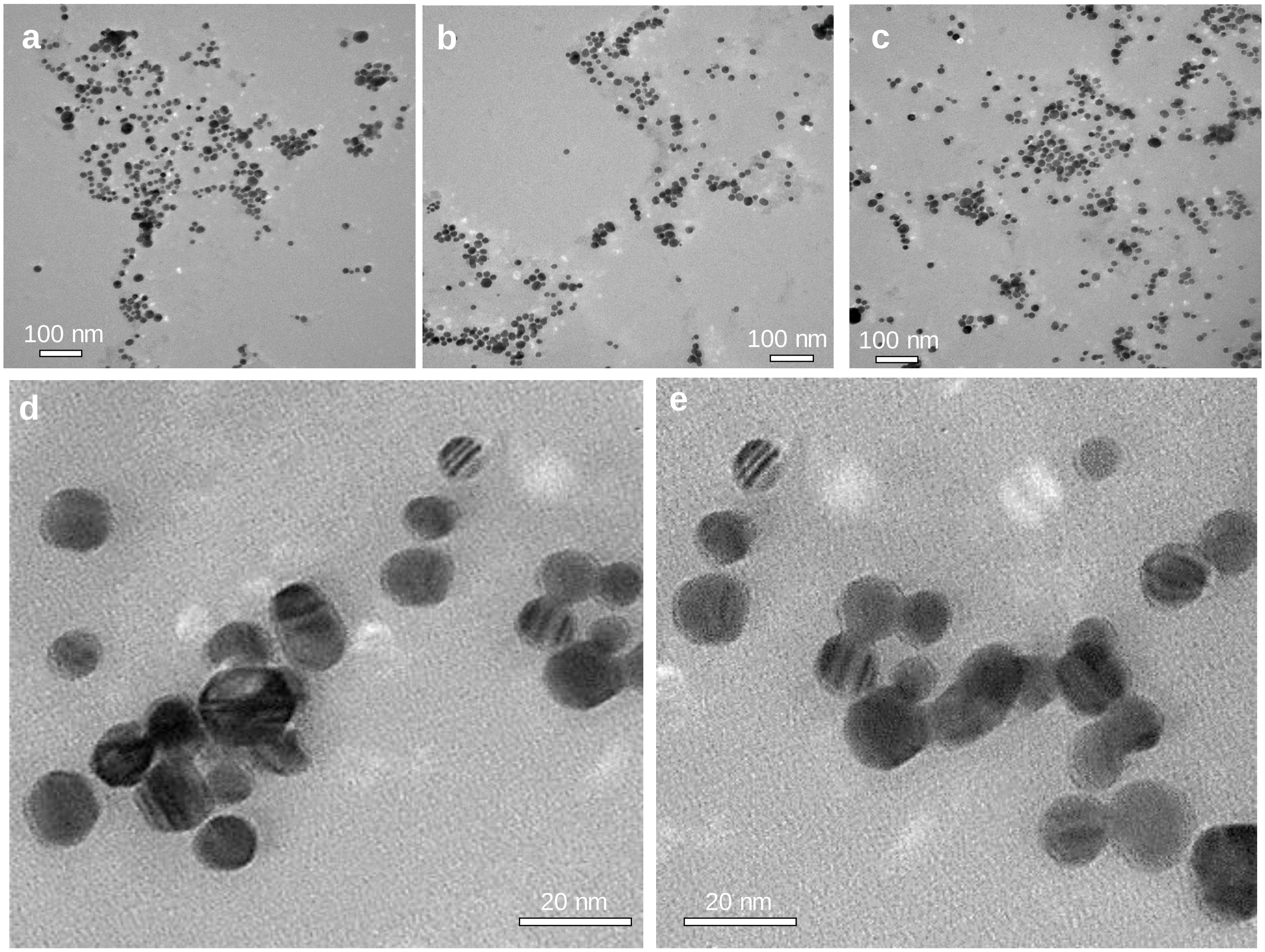

Figure S4. (a), (b) and (c) TEM images of Au-Ag nanoparticles showing their distribution and morphology. (d-e) High magnification TEM images show highlighting the detailed structure of the nanoparticles.

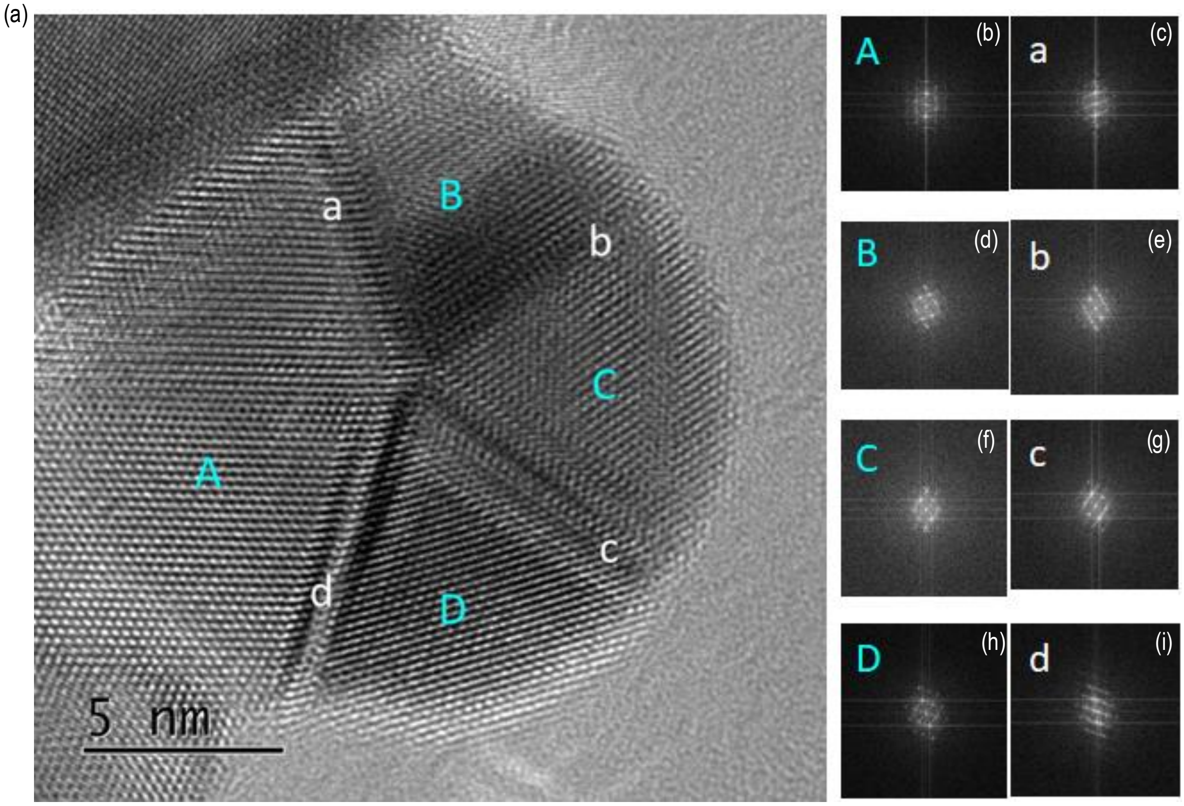

Figure S5. (a). Representative aberration-corrected HRTEM image of Au-Ag core-shell NP. A, B, C, D, a, b, c, and d denote the locations for FFT analysis. (b) – (i) FFT patents probed from A, B, C, D, a, b, c, and d shown in (a). Experimental conditions: 9 mA plasma current, 20 minutes process time, and 0.254 mM $HAuCl_4(_{aq})$.

Note.

To investigate the nanocrystalline configurations of the synthesized Au core, fast Fourier transform (FFT) patterns were observed along the (001) zone axis of the Au core. FFT patterns captured at various locations are shown in **Fig. S6**), indicating the presence of twinning and stacking faults in the Au core.

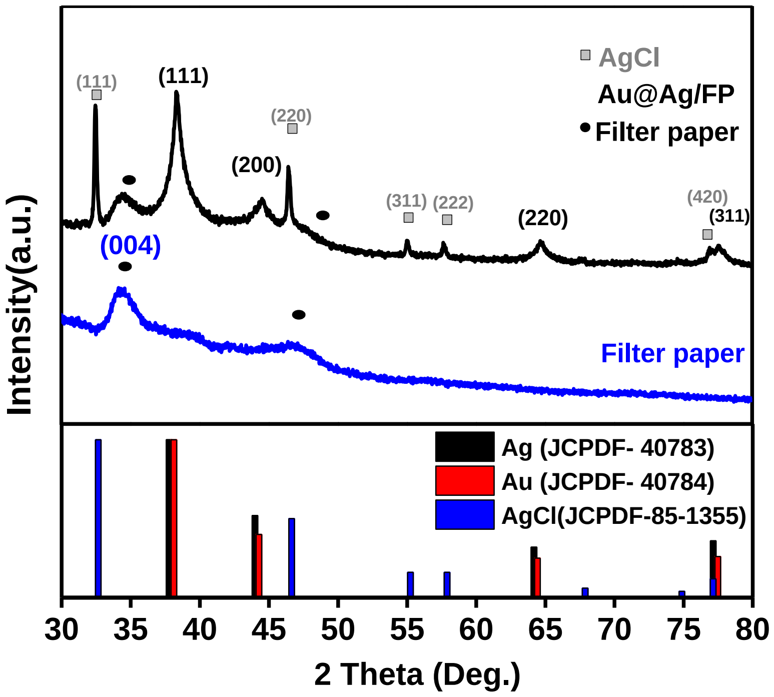

Figure S6. XRD patterns of blank papers and Au-Ag core-shell NPs on cellulose papers. Experimental conditions: 9 mA plasma current, 20 minutes process time, and 0.254 mM $HAuCl_4(_{aq})$.

Note.

Fig. S7 shows the XRD patterns of the as-fabricated Au-Ag core-shell NPs on the paper and blank paper. Scherrer equation (d= Kλ/βcosθ) was used to estimate the average particle size of Au-Ag NPs to be 10 and 21 nm for the Au and Ag phases, respectively, which is consistent with the TEM observations (see TEM discussion).

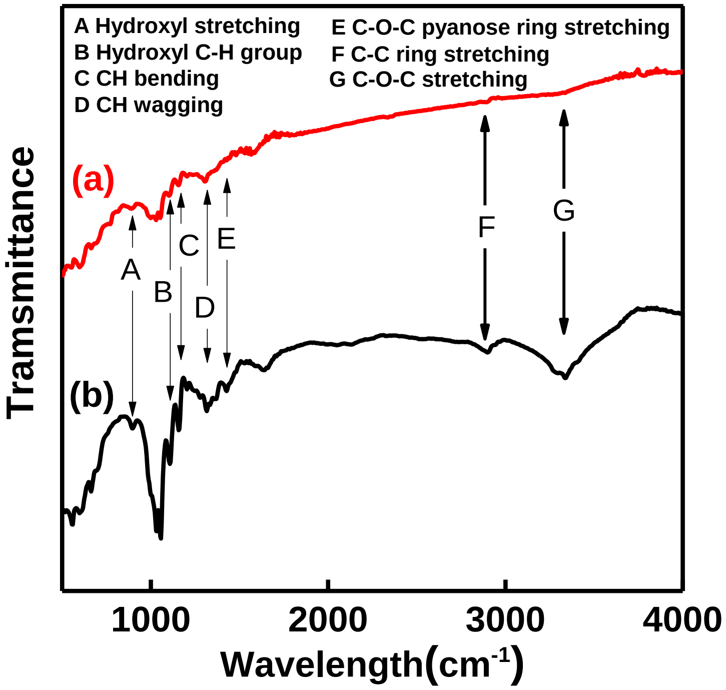

Figure S7. FTIR spectra of blank papers and the Au-Ag core-shell NPs on cellulose papers. Experimental conditions: 9 mA plasma current, 20 minutes process time, and 0.254 mM $HAuCl_4(_{aq})$.

Note

Fig. S8 presents FTIR spectra showing stretching vibrations of the hydroxyl and C-H groups of cellulose, suggesting a hydrophilic sample with a high absorption ability of probe molecules in aqueous solutions for Raman detection.

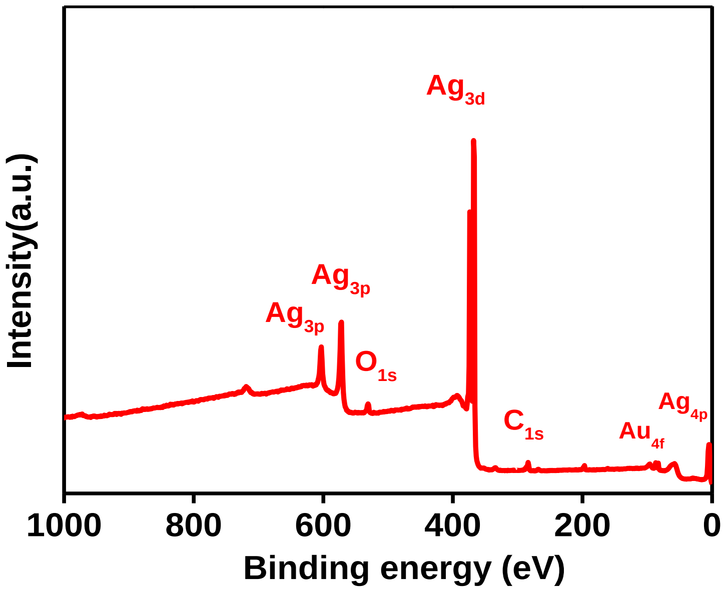

Figure S8. XPS survey scan of Au-Ag core-shell NPs on cellulose paper. Experimental conditions: 9 mA plasma current, 20 minutes process time, and 0.254 mM $HAuCl_4(_{aq})$.

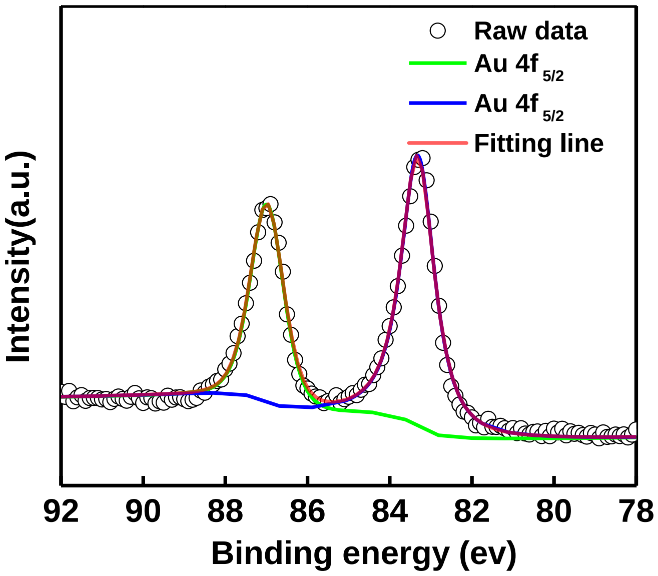

Figure S9. HRXPS Au 4f spectrum of Au Ag core-shell NPs on cellulose paper. Experimental conditions: 9 mA plasma current, 20 minutes process time, and 0.254 mM $HAuCl_4(_{aq})$.

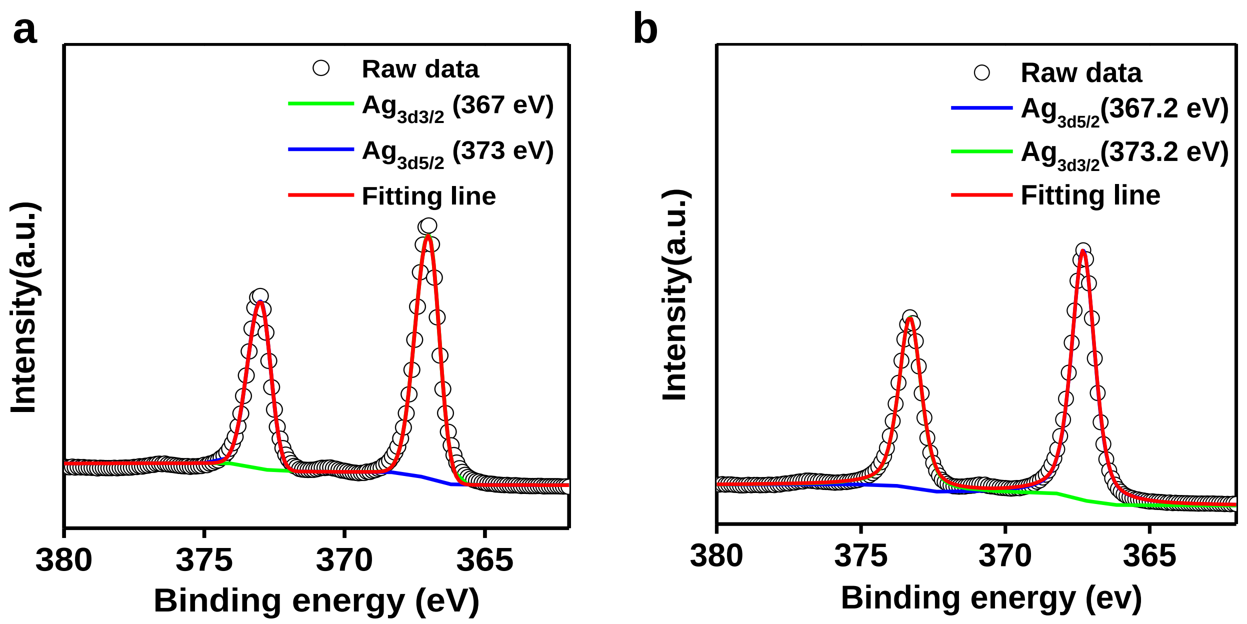

Figure S10. HR-XPS Ag 3d spectra of (a)Ag and (b)Au-Ag core-shell NPs on cellulose papers.

Note.

The charge transfer in Ag and Au–Ag NPs was studied using XPS measurements. High-resolution XPS core-level spectra are shown in **Fig. S10**, focusing on the asymmetrically broadened $3d_{5/2}$ component of Ag. This component exhibited peaks that were effectively separated using Gaussian functions to isolate Ag0 components. Specifically, in pure Ag NPs, the $3d_{5/2}$ component was deconvoluted to reveal an Ag peak at 367.2 eV (**see Fig. S10a**). In contrast, in Au–Ag NPs, this component was discerned at 367 eV (**see Fig. S10b**), illustrating subtle shifts induced by the Au context. The lack of an Ag oxide peak in Au–Ag nanoparticles, despite the exposed Ag shell, highlights their enhanced chemical stability due to the stabilizing electronic charge transfer from Au to Ag, which improves their resistance to oxidation.

XPS was used to examine the compositions and chemical states of the fabricated substrates. The results revealed the presence of metallic states of Au and Ag within the samples **(Fig. S9-10**), which should process the plasmonic properties of the SERS. Moreover, the surface groups of cellulose, including C-OH (285.5 eV) and C-O-C (287.2 eV), were reduced during microplasma synthesis **(Fig. S11-12**).

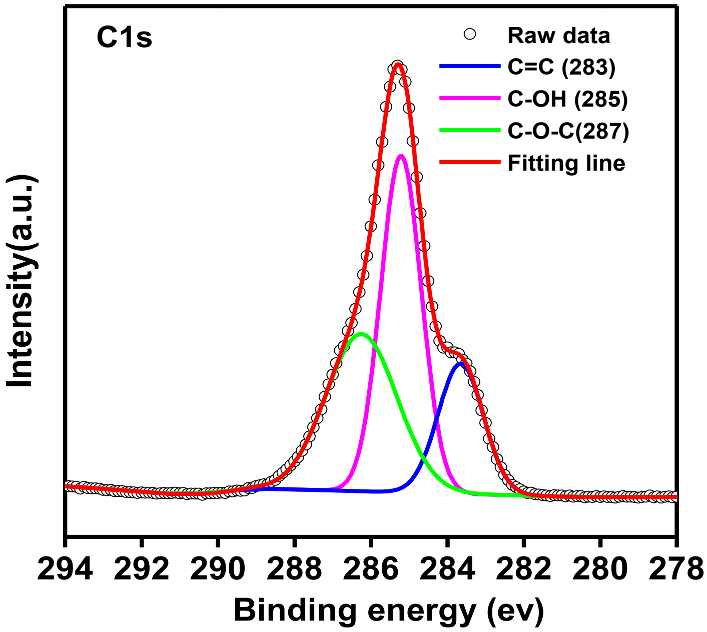

Figure S11. HRXPS C1s spectrum of the blank paper before plasma treatment.

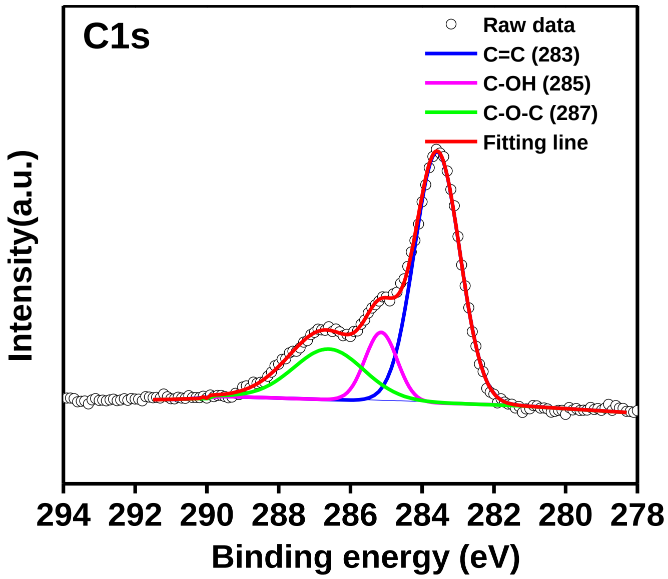

Figure S12. HRXPS C1s spectrum of Au-Ag core-shell NPs on cellulose paper. Experimental conditions: 9 mA plasma current, 20 minutes process time, and 0.254 mM $HAuCl_{4(aq)}$.

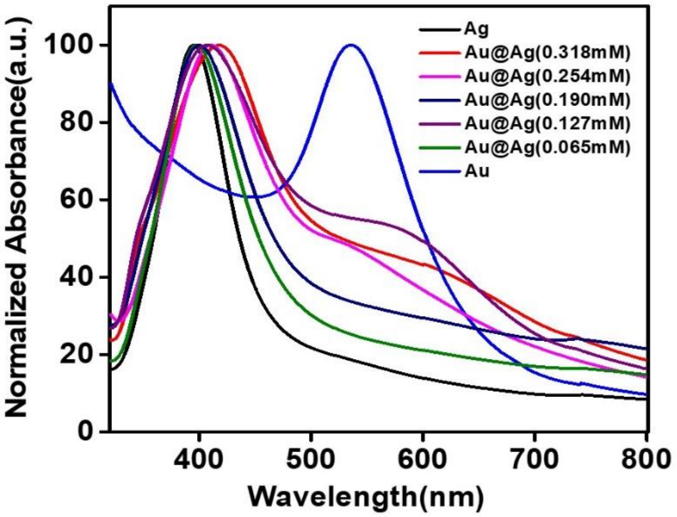

Figure S13. Normalized absorbance spectra for Ag, Au, and Au-Ag core-shell NPs. Au-Ag NPs were prepared from different $HAuCl_4$ concentrations (0.065 ,0.113, 0.127, 0.254 and 0.318mM. The experimental conditions were a plasma current of 9 mA and process time of 20 min.

Note.

The maximization of the SERS effect for AuAgCSNP synthesized at 0.254 mM can be explained by the optimal formation of the core-shell structure, which enhances the localized surface plasmon resonance (LSPR). At 0.254 mM, the balance between the gold core and the silver shell is ideal, creating a sufficient number of plasmonic "hot spots" that significantly amplify the SERS signal. Below this concentration, there may not be enough silver to form a uniform shell, leading to fewer hot spots and a weaker SERS signal. Beyond 0.254 mM, excess silver can cause aggregation of nanoparticles or thicker shells, which may reduce the plasmonic coupling efficiency and thus decrease the SERS effect.

Regarding the shift of the UV-Vis peak of silver to higher wavelengths with increasing precursor concentration, this red shift can be attributed to the growth of silver nanoparticles and the changes in their dielectric environment. As the concentration of the precursor increases, larger nanoparticles or thicker shells form, which results in a change in the local refractive index and an increase in particle size. These factors shift the surface plasmon resonance to longer wavelengths, reflecting the increased particle size and aggregation.

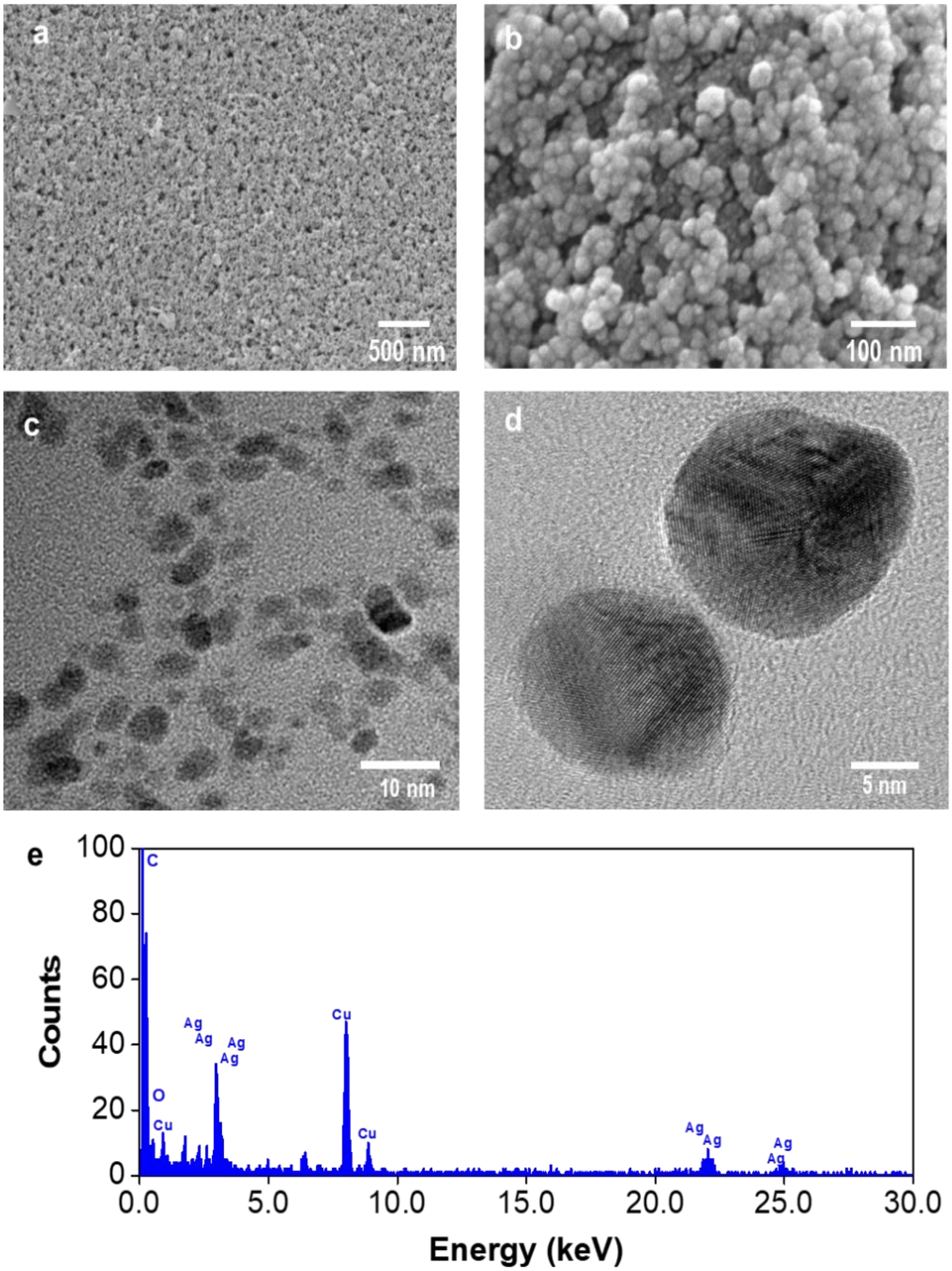

Figure S14. Electron microscopy characterization of The fabricated NPs. (a) Low-and (b) high-magnification SEM images of Ag NPs. (a) Low-and (b) high-magnification TEM images of Ag NPs. (e) TEM-EDX spectrum of Ag NPs.

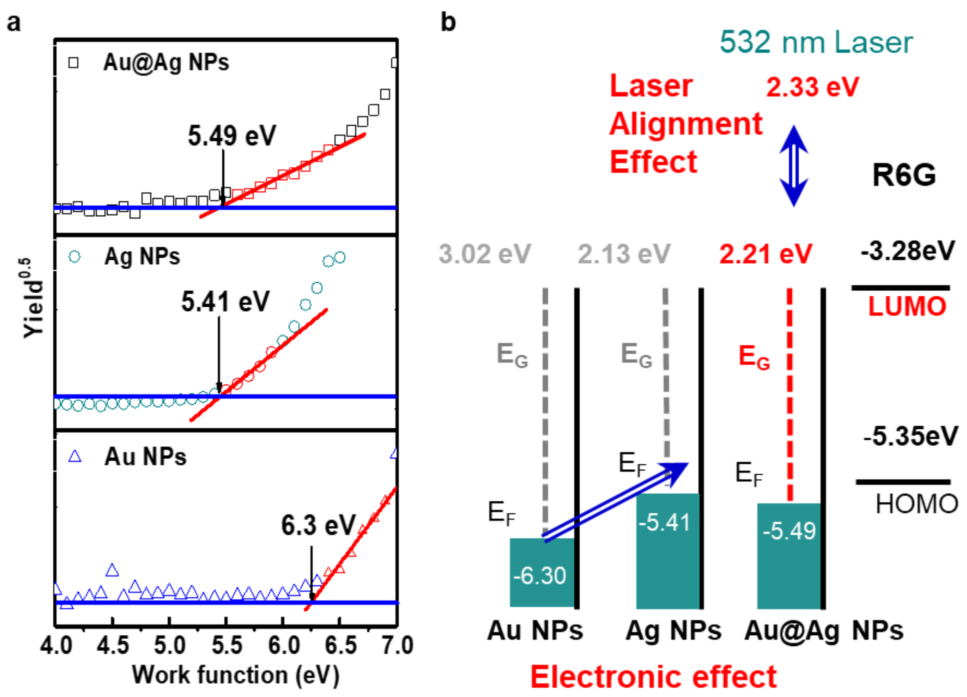

Figure S15 (a) Work function measurements of Au@Ag, Ag, and Au nanoparticles (NPs), showing electronic effects with corresponding energy levels (5.49 eV, 5.41 eV, and 6.3 eV). (b) Schematic of laser alignment and electronic effects illustrating the energy band alignments and resonance enhancement under 532 nm laser excitation.

Note.

The importance of considering the resonance Raman properties of Rhodamine 6G (R6G) under 532 nm excitation. The choice of the 532 nm laser wavelength is grounded in its effective alignment with the electronic transitions of R6G, specifically between the highest occupied molecular orbital (HOMO) and the lowest unoccupied molecular orbital (LUMO). The laser energy at 532 nm closely matches the electronic transition energies of R6G, creating a resonance condition that significantly enhances the Raman scattering cross-section and, consequently, the signal intensity. This phenomenon, known as resonance Raman enhancement, allows for more efficient excitation and improved sensitivity in detecting R6G molecules. Previous studies have demonstrated that selecting an excitation wavelength that aligns with the electronic transition energies of the target molecule, such as 532 nm for R6G, optimizes the Raman signal due to the increased resonance effect[18]. Therefore, we employed a 532 nm laser in our experiments to achieve optimal resonance Raman conditions, facilitating enhanced detection capabilities for R6G.

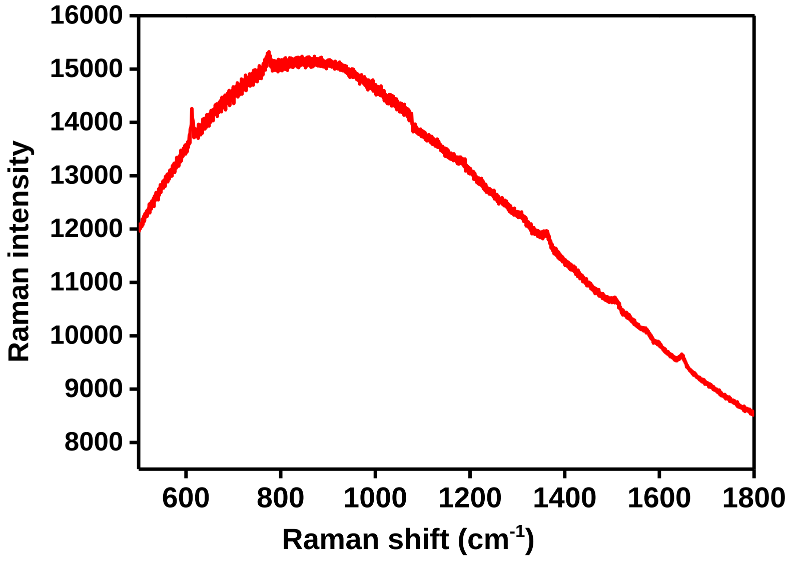

Figure S16. Raman spectrum of the filter paper with 10^{-3}M R6G.

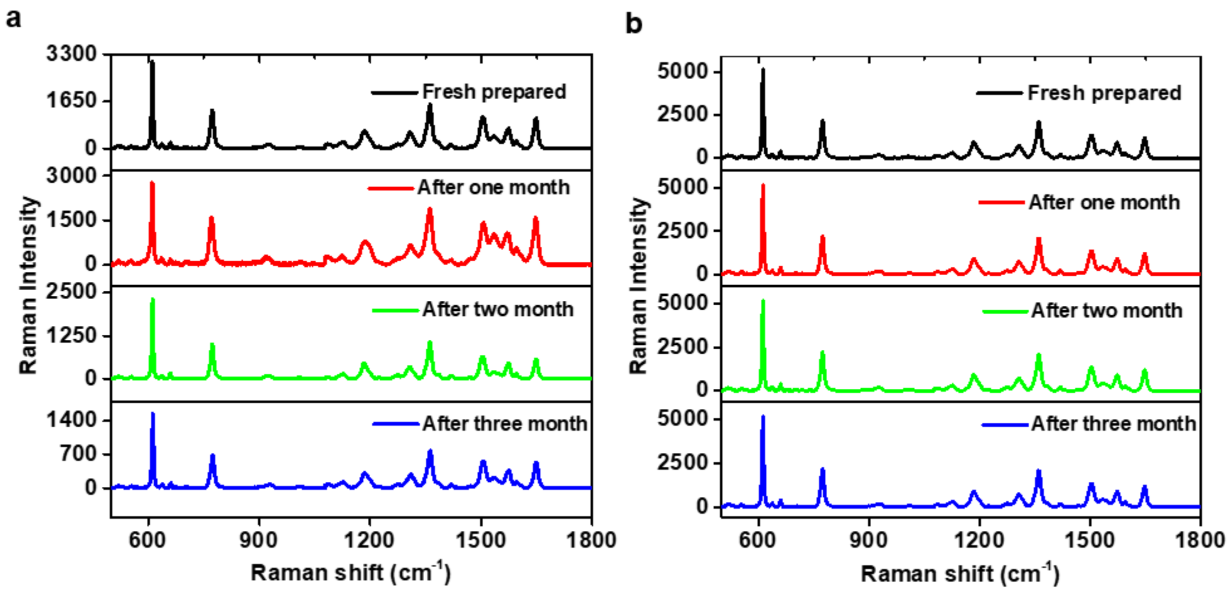

Figure S17. Time-stability study of averaged SERS spectra of 10^{-4} M R6G collected from (a) Ag and (b) Au-Ag core-shell NPs.

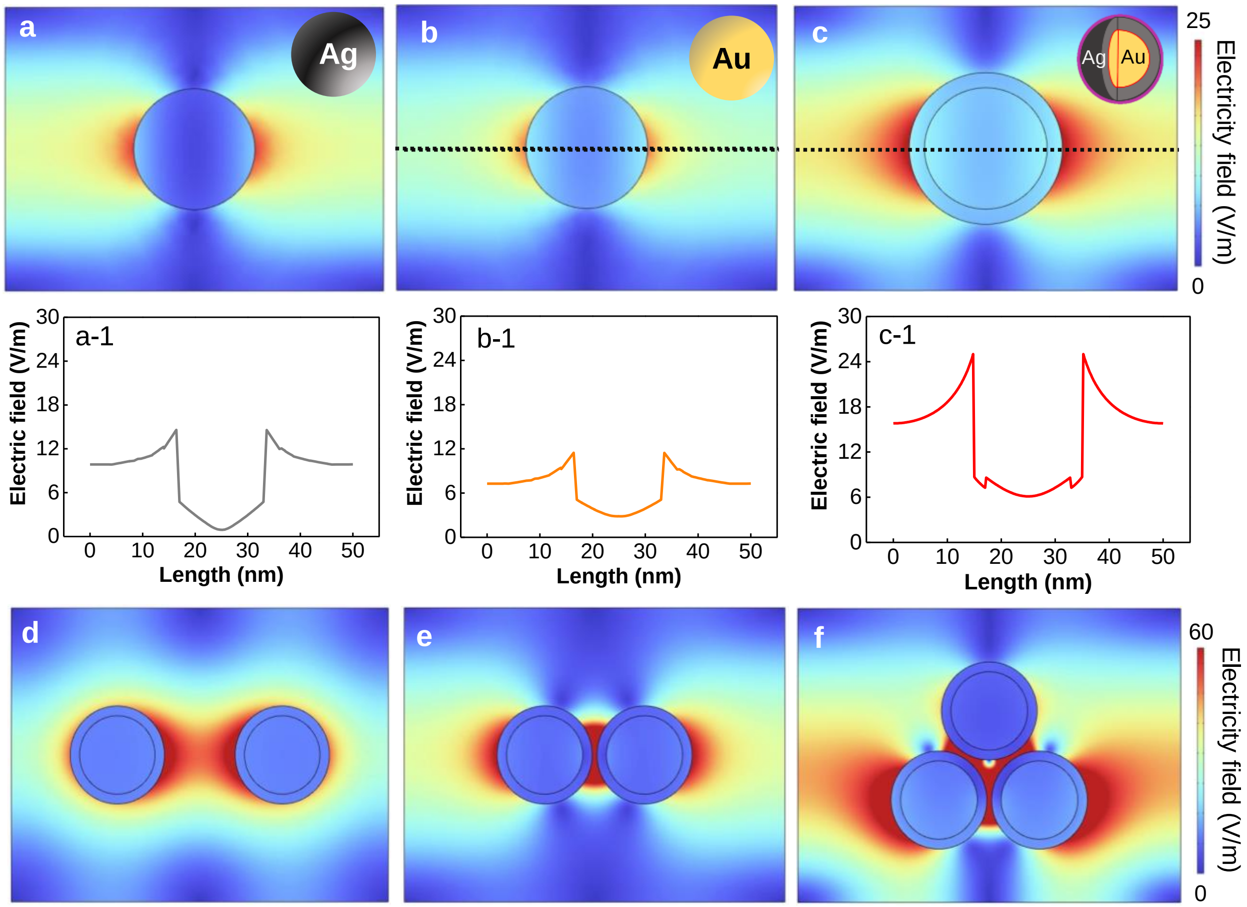

Figure S18. FDTD electric-field distributions for various plasmonic nanostructures under excitation at 532 nm. FDTD electric field distributions for (a) Ag, (b) Au, and (c) Au-Ag core-shell NPs. (a-1), (b-1), and (c-1) show the profiles of the electric field along the Ag, (b) Au, and (c) Au-Ag core-shell NPs, respectively. FDTD electric field distributions for (d) Au-Ag core-shell NPs with a 15 nm gap, (b) Au-Ag core-shell NPs with a 1 nm gap, and (c) stacked Au-Ag core-shell NPs with a 1 nm gap.

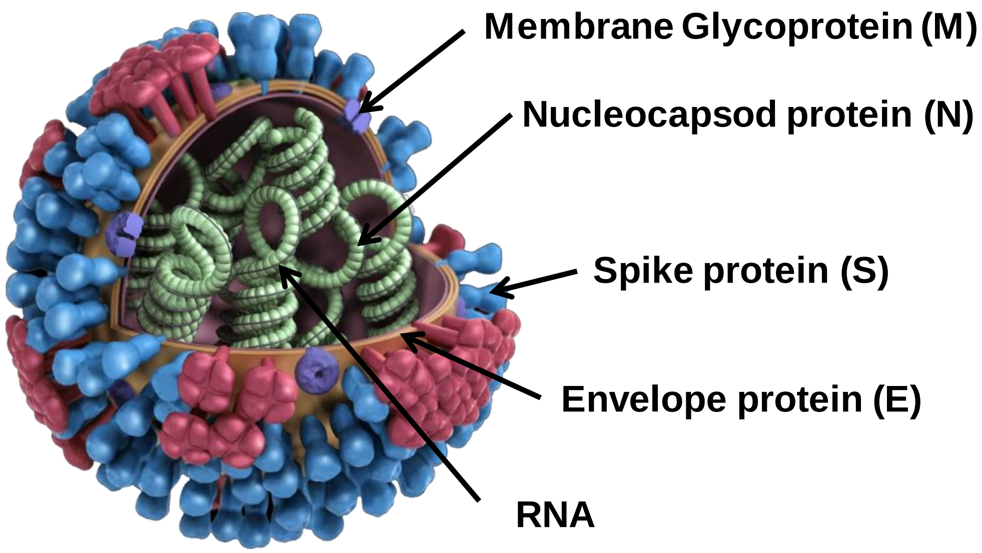

Figure S19. Schematic representation of the SARS-CoV-2 virus structure. The viral RNA is enclosed within the nucleocapsid proteins (N proteins). Surrounding the RNA are the envelope proteins (E proteins), membrane glycoproteins (M), and spike proteins (S proteins). S proteins (S) protrude from the virus surface and play a critical role in viral entry into host cells.

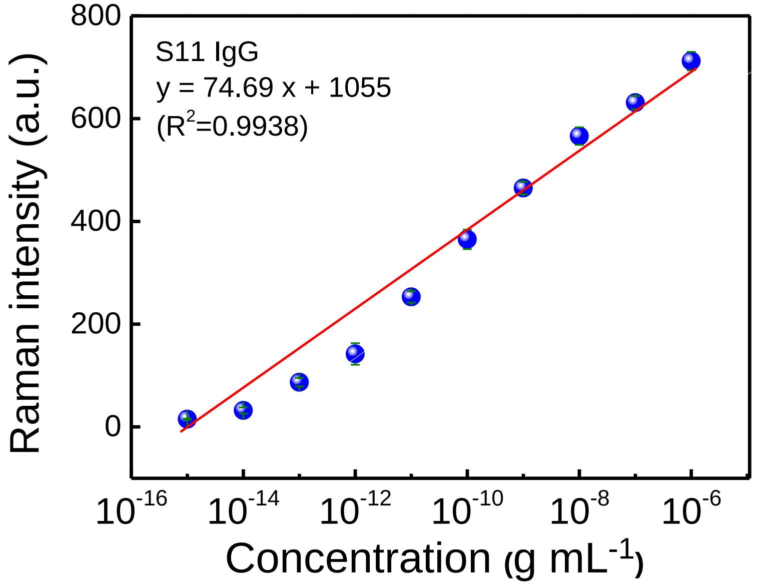

Figure S20. SERS calibration line for SARS-CoV-2 S proteins: the corresponding calibration curve of the peak intensity at 2946 cm^{-1}.

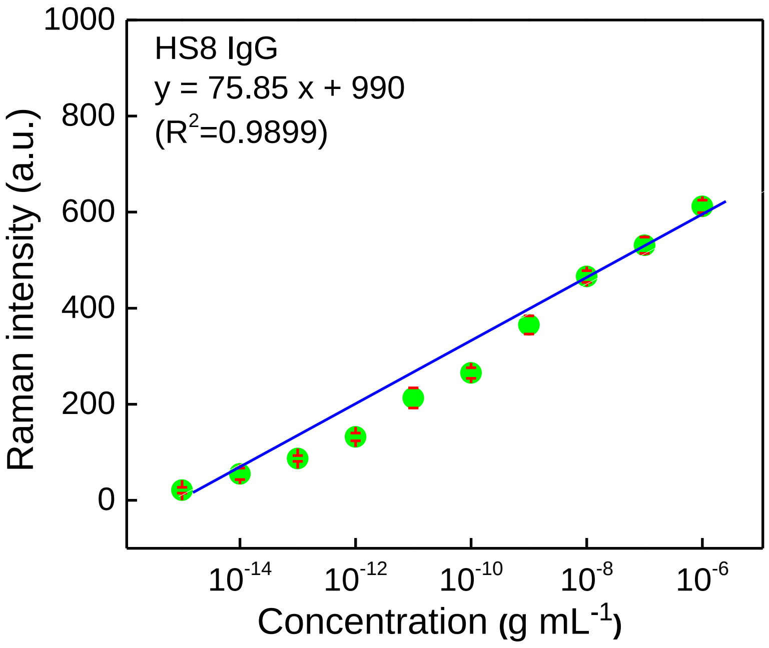

Figure S21. SERS calibration lines for SARS-CoV-2 N proteins and the corresponding calibration curve of peak intensity at 2946 cm^{-1}.

Table S1. Comparision of differnt SERS-active materialsg for **R6G** SERS detection

Analyte	SERS platform	EF	LOD (M)	Ref.
**Rhodamine6G**	**Au-Ag core-shell NP-based 3D HS**	**~1** x $\mathbf{10^{15}}$	**1** x $\mathbf{10^{-15}}$	This work
	Au nanorod	10^6	10^{-9}	19
	Au-Ag biNP/MoS_2	9.67 x 10^9	10^{-13}	20
	Ag nanocube	2 x 10^6	10^{-9}	21
	Ag-decorated Si micropillar array	1.5 x 10^6	10^{-17}	22
	Ag coated natural taro-leaf	10^6	10^{-6}	23
	Array of Ag nanocubes	10^{11}	10^{-10}	24
	Slippery liquid-infused porous Au NP	N.A.	10^{-13}	25
	Ag octahedron@graphene oxide	9.8 x 10^5	10^{-10}	26
	Au NP , Ag NP	1.3 x 10^8	10^{-7}	27
	Graphene oxide attached to Au nanopopcorn	3.8 x 10^{11}	10^{-10}	28
	Ag deposited polycarbonate film	4.9 x 10^6	10^{-7}	29
	Cu_2O mesopous sphere	8 x 10^5	10^{-9}	30
	$MoS_{2-x}O_y$	1.1 x 10^4	10^{-7}	31
	MoO_2	3.75 x 10^6	10^{-7}	32
	Graphene quantum dot	2.37 x 10^3	10^{-9}	33

Table S2. Characteristic Raman peaks of SARS-CoV-2 S proteins with the assignment.

**Raman vibration mode**	**Raman shift (cm^{-1})**
C−S stretching	632
C-C bond stretching	725
C−H stretching or N−H deformation	793
CH_2 rocking or phenylalanine out-of-plane bending	892
α-helical skeletal vibration	946
NH rocking or C−N stretching	1055
Tyrosine vibration	1115
C−C stretching of tyrosine and phenylalanine, tryptophan rocking	1267
Amide III α-helix vibration	1283
Tryptophan C_α-H deformation	1339
CH_3 symmetric stretching	1369
COO- symmetric stretching	1467
NH_3^+ deformation of lysine	1526
Tryptophan aromatic ring stretching	1580
Phenylalanine or tyrosine C−C ring stretching	1601
CH stretching	2895
CH_3 stretching	2946
OH stretching	3481

Table S3.Comparison of SERS technology *with* currently applied methods for SARS-CoV-2 detection[16].

	**SERS (This work) (Au-Ag core-shell NP-based 3DHS)**	**Nucleic acid-based test**	**Serological test**
Analyte(s)	Viral envelope and membrane proteins	Viral RNA	Antibody/antigen
Acquisition time	Less than 1 min	15 min – 8 h	15 – 30 min
Timeframe of effectivity	No limit	Early infection stage (6 days before to 14 days after symptom onset)	Late infection stage (7 days after symptom onset)
Selectivity	Depends upon the condition – some substrates have component with high affinity to the target, thus resulting to high selectivity	High specific; targets viral RNA specific to a particular virus	Low; can produce false positives with other same category viruses
Estimated cost per test	10 USD	100 – 300 USD	25 – 100 USD

Table S4. Performance comparison of different COVID-19 sensors.

Method	Analyte	LOD	Sample	Ref.
**SERS-based biosensor (Au-Ag cre-shell NP-based 3DHS)**	**Spike protein**	**100.0 ag mL^{-1}**	**Simulated Human saliva**	**This work**
	**Nucleocapsid Protein**	**1 fg mL^{-1}**	**Simulated Human saliva**	
SERS flow assay (goldNanostar)	Spike protein	100 fg mL^{-1}	-	34
SERS flow immunoassay	Spike protein	0.1 ng mL^{-1}	-	35
SERS-based biosensor	Inactivated whole virus	5.5×10^4 TCID 50/mL	-	36
SERS-based biosensor	Nucleocapsid Protein	0.5 pM		37
SERS-based biosensor	Spike protein	300 nM		38
SLIP- SERS	Spike protein	1 pM	-	39
Nanozyme-based chemiluminescence paper assay	Spike protein	0.1 ng mL^{-1}	-	40
Lateral flow immunoassay (LFIA)-based biosensor	Nucleocapsid Protein	2.5×10^4 pfu/reaction	-	41
Electrochemical Immunosensor	Spike protein	19 ng mL^{-1}	Untreated saliva	42
Dual-functional plasmonic photothermal biosensors	-	0.22 pM	Unamplified nucleic acid	43
FET biosensor	Spike protein	100 fg mL^{-1}	Nasopharyngeal swabs	44
Terahertz plasmonic biosensors	Spike protein	~4.2 fM	PBS	45
Paper-based electrochemical biosensor	Spike protein	0.11 ng mL^{-1}	-	46

Table S5. Comparison of performance of different **ketoprofen** sensors

Analyte technique	Material	Linear range	LOD	Ref.
**SERS**	**Au-Ag cre-shell NP-based 3DHS**	**10^{-3} – 10^{-10} (M)**	**10^{-10} (M) ($EF\sim10^{7}$)**	This work
	Silver nanoflower	2-15(mgL^{-1})	1.1mgL^{-1} (EF=1.97x10^{4})	47
PS-MS	Ny-AgNF	0.023-0.046(mgL^{-1})	0.023mgL^{-1}	
Adsorptive Stripping SquareWave	Mercury	1x10^{-8} – 3x10^{-7} (M)	0.1ng mL^{-1}	48
IC-FLD	SnO_2 nanoparticles	0.1 µg/kg	0.2-1.5µg/kg	49
Differential Pulse Polarography	Dropping-mercury electrode	1x10^{-5} – 5x10^{-4}(M)	9.8 x 10^{-6} mol/L	50
Polarography	Dropping-mercury electrode	10^{-8} – 10^{-6}(M)	2.0 x 10^{-9} mol/L	
Differential Pulse Voltammetry	glassy carbon electrode	70 pM^{-6} µM	20pM	51
ITO-LMR probe	ITO electrode	10^{-6}– 10^{-3} (M)	0.5 x 10^{-3} mol/L	52
Stripping voltammetry	Mercury electrode	10^{-8} – 10^{-7}(M)	2.0 x 10^{-9}mol/L	53

Table S6. Comparison of performance of different **folic acid** SERS sensors

Analyte	SERS platform	EF	LOD(M)	Ref.
Folic acid	**Au-Ag cre-shell NP-based 3DHS**	**~10^6**	**10^{-9}**	This work
	AgNPs/MIL-101(Cr)	5.2 x 10^3	$5x10^{-7}$	54
	Mtallic nanotube arrays	4.1 x 10^6	10^{-11}	55
	GO/PDDA/Ag NPs	N.A.	$9x10^{-9}$	56

Table S7 . Comparison of performance of different **folic acid** sensors

Analyte technique	Material	Linear range	LOD	Ref.
High-performance liquid chromatography (HPLC)	·-	123-135(μg/100g)	8.2(μg/100g)	57
	-	61.4-161.6(μg/100g)	-	58
Ezyme-linked immunosorbent assay (ELISA)	BSA	15-82(ng/mL)	-	59
Electrochemical assays (EC)	PPy–α-POM–AuNPs	80nM-10mM	20nM	60
	6-thioguanine	0.70 and 11.0 μmol L^{-1}	-	61
Capillary electrophoresis (CE)	-	1.2×10^{-5} - 4.8×10^{-5} M	6.12×10^{-7} M	62
Fluorescent	Carbon dots	1-300(μM)	0.28	63
	$Pd_{0.05}Ag_{0.1}$ SnO_2/Pt electrode	22-112(μM)	3.47	64

Table S8. Comparison of performance of different **salicylic acid** SERS sensors

Analyte	SERS platform	EF	LOD(M)	Ref.
Salicylic acid	**Au-Ag core-shell NP-based 3DHS**	**10^{6}**	**10^{-9}**	This work
	Ag-Cu-grid substrate	6.1 x10^{5}	6 x10^{-7}	65
	bis (benzoic acid) and Ag nanoparticles (AgNPs)	-	10^{-5}	66
	Ag@MIP	-	3 x 10^{-7}	67
	Ag@PVP		3x10^{-6}	

Table S9. Comparison of previous method for detection **SA** with the present work

Analyte technique	Material	Linear range	LOD	Ref.
Direct voltammetric	rGO	$10^{−10}$ -1.0 × $10^{−5}$ M	2.3 × $10^{−11}$ M	68
HPLC	C18@GO@PDDA	10–5000 μg $g^{−1}$	1.8–2.8 μg $g^{−1}$	69
Dfferential pulse voltammetry	$NiTiO_3$ nanoceramics	40.0–1000.0 μM	68.0 nM	70
Fuorescence spectrometry	PPy/nitrate	5–100 nanomol $L^{−1}$	1.4 nanomol $L^{−1}$	71

Table S10. Molecular Properties of Ketoprofen (KP), Folic Acid (FA), and Salicylic Acid (SA). The table includes molecular weight, dimensions (length, width, height), molecular volume, and molecular surface area for each molecule[72]. These properties influence the adsorption behavior and detection sensitivity in the SERS-based system.

**Chemical**	Ketoprofen (KP)	Folic Acid(FA)	Salicylic Acid (SA)
**Molecular Weight (g/mol)**	254.29	441.40	138.12
**Molecular Dimensions**			
Length (Å)	12-14	15-18	7-8
Width (Å)	6-8	6-8	5-6
Height (Å)	2.5-3.5	3-4	2-3
**Molecular Volume (Å^3)**	230-270	400-450	120-150
**Molecular Surface Area (Å^2)**	220-280	350-400	120-150

Note.

The differences in detection ranges are due to the distinct molecular properties of KP, folic acid (FA), and salicylic acid (SA), such as their molecular weights, dimensions, and surface areas, which affect their interactions with the AuAg3DHS system.Ketoprofen (KP) has a moderate molecular weight and smaller surface area (220-280 Å^2), resulting in fewer interaction sites and a narrower detection range. Folic acid (FA), with a larger molecular weight and surface area (350-400 Å^2), has more functional groups available for adsorption, leading to a broader detection range. Salicylic acid (SA), being the smallest molecule with the lowest surface area (120-150 Å^2), shows a moderate detection range.

The two fitting lines for KP reflect different adsorption behaviors at varying concentrations. At low concentrations (10^{-10} to 10^{-6} M), KP molecules are sparsely distributed, enhancing the SERS signal significantly, resulting in a steep slope. At higher concentrations (10^{-6} to 10^{-4} M), the nanoparticle surface becomes saturated, reducing the signal increase and leading to a second, flatter linear fit.

Reference

1. Kulkarni, A. A.; Bhanage, B. M., Ag@ AgCl nanomaterial synthesis using sugar cane juice and its application in degradation of azo dyes. *ACS Sustainable Chemistry & Engineering* **2014,** *2* (4), 1007-1013.

2. Carrillo, F.; Colom, X.; Sunol, J.; Saurina, J., Structural FTIR analysis and thermal characterisation of lyocell and viscose-type fibres. *European Polymer Journal* **2004,** *40* (9), 2229-2234.

3. Han, S. W.; Kim, Y.; Kim, K., Dodecanethiol-derivatized Au/Ag bimetallic nanoparticles: TEM, UV/VIS, XPS, and FTIR analysis. *Journal of colloid and interface science* **1998,** *208* (1), 272-278.

4. Kruse, N.; Chenakin, S., XPS characterization of Au/TiO2 catalysts: binding energy assessment and irradiation effects. *Applied Catalysis A: General* **2011,** *391* (1-2), 367-376.

5. Shankar, S. S.; Rai, A.; Ahmad, A.; Sastry, M., Rapid synthesis of Au, Ag, and bimetallic Au core–Ag shell nanoparticles using Neem (Azadirachta indica) leaf broth. *Journal of colloid and interface science* **2004,** *275* (2), 496-502.

6. Philip, D., Biosynthesis of Au, Ag and Au–Ag nanoparticles using edible mushroom extract. *Spectrochimica Acta Part A: Molecular and Biomolecular Spectroscopy* **2009,** *73* (2), 374-381.

7. Yilmaz, M.; Babur, E.; Ozdemir, M.; Gieseking, R. L.; Dede, Y.; Tamer, U.; Schatz, G. C.; Facchetti, A.; Usta, H.; Demirel, G., Nanostructured organic semiconductor films for molecular detection with surface-enhanced Raman spectroscopy. *Nature materials* **2017,** *16* (9), 918-924.

8. Kneipp, K.; Wang, Y.; Kneipp, H.; Perelman, L. T.; Itzkan, I.; Dasari, R. R.; Feld, M. S., Single molecule detection using surface-enhanced Raman scattering (SERS). *Physical review letters* **1997,** *78* (9), 1667.

9. Baumberg, J. J.; Aizpurua, J.; Mikkelsen, M. H.; Smith, D. R., Extreme nanophotonics from ultrathin metallic gaps. *Nature materials* **2019,** *18* (7), 668-678.

10. Langer, J.; Jimenez de Aberasturi, D.; Aizpurua, J.; Alvarez-Puebla, R. A.; Auguié, B.; Baumberg, J. J.; Bazan, G. C.; Bell, S. E.; Boisen, A.; Brolo, A. G., Present and future of surface-enhanced Raman scattering. *ACS nano* **2019,** *14* (1), 28-117.

11. Blackie, E. J.; Le Ru, E. C.; Etchegoin, P. G., Single-molecule surface-enhanced Raman spectroscopy of nonresonant molecules. *Journal of the American Chemical Society* **2009,** *131* (40), 14466-14472.

12. Kallepitis, C.; Bergholt, M. S.; Mazo, M. M.; Leonardo, V.; Skaalure, S. C.; Maynard, S. A.; Stevens, M. M., Quantitative volumetric Raman imaging of three dimensional cell cultures. *Nature Communications* **2017,** *8*.

13. Lauri, A.; Velleman, L.; Xiao, X. F.; Cortes, E.; Edel, J. B.; Giannini, V.; Rakovich, A.; Maier, S. A., 3D Confocal Raman Tomography to Probe Field Enhancements inside Supercluster Metamaterials. *Acs Photonics* **2017,** *4* (8), 2070-2077.

14. Sethuraman, N.; Jeremiah, S. S.; Ryo, A., Interpreting diagnostic tests for SARS-CoV-2. *Jama* **2020,** *323* (22), 2249-2251.

15. Wechselberger, C.; Süßner, S.; Doppler, S.; Bernhard, D., Performance evaluation of serological assays to determine the immunoglobulin status in SARS-CoV-2 infected patients. *Journal of Clinical Virology* **2020,** *131*, 104589.

16. Sitjar, J.; Der-Liao, J.; Lee, H.; Tsai, H.-P.; Wang, J.-R.; Liu, P.-Y., Challenges of SERS technology as a non-nucleic acid or-antigen detection method for SARS-CoV-2 virus and its variants. *Biosensors Bioelectron.* **2021**, 113153.

17. Cennamo, N.; Pasquardini, L.; Arcadio, F.; Lunelli, L.; Vanzetti, L.; Carafa, V.; Altucci, L.; Zeni, L., SARS-CoV-2 spike protein detection through a plasmonic D-shaped plastic optical fiber aptasensor. *Talanta* **2021,** *233*, 122532.

18. Hang, Y.; Boryczka, J.; Wu, N., Visible-light and near-infrared fluorescence and surface-enhanced Raman scattering point-of-care sensing and bio-imaging: A review. *Chemical Society Reviews* **2022,** *51* (1), 329-375.

19. Kim, K.; Han, H. S.; Choi, I.; Lee, C.; Hong, S.; Suh, S.-H.; Lee, L. P.; Kang, T., Interfacial liquid-state surface-enhanced Raman spectroscopy. *Nature communications* **2013,** *4* (1), 1-9.

20. Xu, J.; Li, C.; Si, H.; Zhao, X.; Wang, L.; Jiang, S.; Wei, D.; Yu, J.; Xiu, X.; Zhang, C., 3D SERS substrate based on Au-Ag bi-metal nanoparticles/MoS 2 hybrid with pyramid structure. *Optics express* **2018,** *26* (17), 21546-21557.

21. Phan-Quang, G. C.; Lee, H. K.; Phang, I. Y.; Ling, X. Y., Plasmonic colloidosomes as three-dimensional SERS platforms with enhanced surface area for multiphase sub-microliter toxin sensing. *Angewandte Chemie* **2015,** *127* (33), 9827-9831.

22. De Angelis, F.; Gentile, F.; Mecarini, F.; Das, G.; Moretti, M.; Candeloro, P.; Coluccio, M.; Cojoc, G.; Accardo, A.; Liberale, C., Breaking the diffusion limit with super-hydrophobic

delivery of molecules to plasmonic nanofocusing SERS structures. *Nature Photonics* **2011,** *5* (11), 682-687.
23. Huang, J.-A.; Zhang, Y.-L.; Zhao, Y.; Zhang, X.-L.; Sun, M.-L.; Zhang, W., Superhydrophobic SERS chip based on a Ag coated natural taro-leaf. *Nanoscale* **2016,** *8* (22), 11487-11493.
24. Lee, H. K.; Lee, Y. H.; Zhang, Q.; Phang, I. Y.; Tan, J. M. R.; Cui, Y.; Ling, X. Y., Superhydrophobic surface-enhanced Raman scattering platform fabricated by assembly of Ag nanocubes for trace molecular sensing. *ACS applied materials & interfaces* **2013,** *5* (21), 11409-11418.
25. Yang, S.; Dai, X.; Stogin, B. B.; Wong, T.-S., Ultrasensitive surface-enhanced Raman scattering detection in common fluids. *Proceedings of the National Academy of Sciences* **2016,** *113* (2), 268-273.
26. Fan, W.; Lee, Y. H.; Pedireddy, S.; Zhang, Q.; Liu, T.; Ling, X. Y., Graphene oxide and shape-controlled silver nanoparticle hybrids for ultrasensitive single-particle surface-enhanced Raman scattering (SERS) sensing. *Nanoscale* **2014,** *6* (9), 4843-4851.
27. Ben-Jaber, S.; Peveler, W. J.; Quesada-Cabrera, R.; Cortés, E.; Sotelo-Vazquez, C.; Abdul-Karim, N.; Maier, S. A.; Parkin, I. P., Photo-induced enhanced Raman spectroscopy for universal ultra-trace detection of explosives, pollutants and biomolecules. *Nature communications* **2016,** *7* (1), 1-6.
28. Fan, Z.; Kanchanapally, R.; Ray, P. C., Hybrid graphene oxide based ultrasensitive SERS probe for label-free biosensing. *The Journal of Physical Chemistry Letters* **2013,** *4* (21), 3813-3818.
29. Daglar, B.; Khudiyev, T.; Demirel, G. B.; Buyukserin, F.; Bayindir, M., Soft biomimetic tapered nanostructures for large-area antireflective surfaces and SERS sensing. *Journal of Materials Chemistry C* **2013,** *1* (47), 7842-7848.
30. Lin, J.; Shang, Y.; Li, X.; Yu, J.; Wang, X.; Guo, L., Ultrasensitive SERS detection by defect engineering on single Cu2O superstructure particle. *Advanced Materials* **2017,** *29* (5), 1604797.
31. Zheng, Z.; Cong, S.; Gong, W.; Xuan, J.; Li, G.; Lu, W.; Geng, F.; Zhao, Z., Semiconductor SERS enhancement enabled by oxygen incorporation. *Nature communications* **2017,** *8* (1), 1-10.

32. Zhang, Q.; Li, X.; Ma, Q.; Zhang, Q.; Bai, H.; Yi, W.; Liu, J.; Han, J.; Xi, G., A metallic molybdenum dioxide with high stability for surface enhanced Raman spectroscopy. *Nature communications* **2017,** *8* (1), 1-9.

33. Liu, D.; Chen, X.; Hu, Y.; Sun, T.; Song, Z.; Zheng, Y.; Cao, Y.; Cai, Z.; Cao, M.; Peng, L., Raman enhancement on ultra-clean graphene quantum dots produced by quasi-equilibrium plasma-enhanced chemical vapor deposition. *Nature communications* **2018,** *9* (1), 1-10.

34. Srivastav, S.; Dankov, A.; Adanalic, M.; Grzeschik, R.; Tran, V.; Pagel-Wieder, S.; Gessler, F.; Spreitzer, I.; Scholz, T.; Schnierle, B., Rapid and Sensitive SERS-Based Lateral Flow Test for SARS-CoV2-Specific IgM/IgG Antibodies. *Analytical Chemistry* **2021,** *93* (36), 12391-12399.

35. Chen, S.; Meng, L.; Wang, L.; Huang, X.; Ali, S.; Chen, X.; Yu, M.; Yi, M.; Li, L.; Chen, X., SERS-based lateral flow immunoassay for sensitive and simultaneous detection of anti-SARS-CoV-2 IgM and IgG antibodies by using gap-enhanced Raman nanotags. *Sensors and Actuators B: Chemical* **2021,** *348*, 130706.

36. Zavyalova, E.; Ambartsumyan, O.; Zhdanov, G.; Gribanyov, D.; Gushchin, V.; Tkachuk, A.; Rudakova, E.; Nikiforova, M.; Kuznetsova, N.; Popova, L., SERS-Based Aptasensor for Rapid Quantitative Detection of SARS-CoV-2. *Nanomaterials* **2021,** *11* (6), 1394.

37. Ji, Z.; Zhang, C.; Ye, Y.; Ji, J.; Dong, H.; Forsberg, E.; Cheng, X.; He, S., Magnetically enhanced liquid SERS for ultrasensitive analysis of bacterial and SARS-Cov-2 biomarkers. *Frontiers in bioengineering and biotechnology* **2021,** *9*.

38. Payne, T. D.; Klawa, S. J.; Jian, T.; Kim, S. H.; Papanikolas, M. J.; Freeman, R.; Schultz, Z. D., Catching COVID: Engineering Peptide-Modified Surface-Enhanced Raman Spectroscopy Sensors for SARS-CoV-2. *ACS sensors* **2021,** *6* (9), 3436-3444.

39. Stanborough, T.; Given, F. M.; Koch, B.; Sheen, C. R.; Stowers-Hull, A. B.; Waterland, M. R.; Crittenden, D. L., Optical Detection of CoV-SARS-2 Viral Proteins to Sub-Picomolar Concentrations. *ACS omega* **2021,** *6* (9), 6404-6413.

40. Liu, D.; Ju, C.; Han, C.; Shi, R.; Chen, X.; Duan, D.; Yan, J.; Yan, X., Nanozyme chemiluminescence paper test for rapid and sensitive detection of SARS-CoV-2 antigen. *Biosensors and Bioelectronics* **2021,** *173*, 112817.

41. Seo, G.; Lee, G.; Kim, M. J.; Baek, S.-H.; Choi, M.; Ku, K. B.; Lee, C.-S.; Jun, S.; Park, D.; Kim, H. G., Rapid detection of COVID-19 causative virus (SARS-CoV-2) in human

nasopharyngeal swab specimens using field-effect transistor-based biosensor. *ACS nano* **2020,** *14* (4), 5135-5142.

42. Fabiani, L.; Saroglia, M.; Galatà, G.; De Santis, R.; Fillo, S.; Luca, V.; Faggioni, G.; D'Amore, N.; Regalbuto, E.; Salvatori, P., Magnetic beads combined with carbon black-based screen-printed electrodes for COVID-19: A reliable and miniaturized electrochemical immunosensor for SARS-CoV-2 detection in saliva. *Biosensors and Bioelectronics* **2021,** *171*, 112686.

43. Worramongkona, P.; Seeda, K.; Phansomboon, P.; Ratnarathorn, N.; Chailapakul, O.; Dungchai, W., A simple paper-based colorimetric device for rapid and sensitive urinary oxalate determinations. *Analytical Sciences* **2018,** *34* (1), 103-108.

44. Fakhari, A. R.; Rafiee, B.; Ahmar, H.; Bagheri, A., Electrocatalytic determination of oxalic acid by TiO 2 nanoparticles/multiwalled carbon nanotubes modified electrode. *Analytical Methods* **2012,** *4* (10), 3314-3319.

45. Akhond, M.; Absalan, G.; Tafakori, A.; Ershadifar, H., Simultaneous determination of thiocyanate and oxalate in urine using a carbon ionic liquid electrode modified with TiO2-Fe nanoparticles. *Analytical and Bioanalytical Chemistry Research* **2016,** *3* (1), 73-86.

46. Yakoh, A.; Pimpitak, U.; Rengpipat, S.; Hirankarn, N.; Chailapakul, O.; Chaiyo, S., based electrochemical biosensor for diagnosing COVID-19: Detection of SARS-CoV-2 antibodies and antigen. *Biosensors and Bioelectronics* **2021,** *176*, 112912.

47. Díaz-Liñán, M.; García-Valverde, M.; López-Lorente, A.; Cárdenas, S.; Lucena, R., Silver nanoflower-coated paper as dual substrate for surface-enhanced Raman spectroscopy and ambient pressure mass spectrometry analysis. *Analytical and bioanalytical chemistry* **2020,** *412* (15), 3547-3557.

48. Ghoneim, M.; Tawfik, A., Voltammetric studies and assay of the anti-inflammatory drug ketoprofen in pharmaceutical formulation and human plasma at a mercury electrode. *Canadian journal of chemistry* **2003,** *81* (8), 889-896.

49. Abdel-Hamid, M. E.; Novotny, L.; Hamza, H., Determination of diclofenac sodium, flufenamic acid, indomethacin and ketoprofen by LC-APCI-MS. *Journal of pharmaceutical and biomedical analysis* **2001,** *24* (4), 587-594.

50. Amankwa, L.; Chatten, L. G., Electrochemical reduction of ketoprofen and its determination in pharmaceutical dosage forms by differential-pulse polarography. *Analyst* **1984,** *109* (1), 57-60.

51. Roushani, M.; Shahdost-Fard, F., Covalent attachment of aptamer onto nanocomposite as a high performance electrochemical sensing platform: Fabrication of an ultra-sensitive ibuprofen electrochemical aptasensor. *Materials Science and Engineering: C* **2016,** *68*, 128-135.

52. Bogdanowicz, R.; Niedziałkowski, P.; Sobaszek, M.; Burnat, D.; Białobrzeska, W.; Cebula, Z.; Sezemsky, P.; Koba, M.; Stranak, V.; Ossowski, T., Optical detection of ketoprofen by its electropolymerization on an indium tin oxide-coated optical fiber probe. *Sensors* **2018,** *18* (5), 1361.

53. Emara, K. M.; Ali, A. M.; Abo-El Maali, N., The polarographic behaviour of ketoprofen and assay of its capsules using spectrophotometric and voltammetric methods. *Talanta* **1994,** *41* (5), 639-645.

54. Sun, Z. J.; Jiang, Z. W.; Li, Y. F., Poly (dopamine) assisted in situ fabrication of silver nanoparticles/metal–organic framework hybrids as SERS substrates for folic acid detection. *RSC advances* **2016,** *6* (83), 79805-79810.

55. Lu, Y.-C.; Chiang, W.-H.; Liu, C.-Y.; Chu, J. P.; Ho, H.-C.; Hsueh, C.-H., Wafer-scale SERS metallic nanotube arrays with highly ordered periodicity. *Sensors and Actuators B: Chemical* **2021,** *329*, 129132.

56. Ren, W.; Fang, Y.; Wang, E., A binary functional substrate for enrichment and ultrasensitive SERS spectroscopic detection of folic acid using graphene oxide/Ag nanoparticle hybrids. *ACS nano* **2011,** *5* (8), 6425-6433.

57. Gujska, E.; Kuncewicz, A., Determination of folate in some cereals and commercial cereal-grain products consumed in Poland using trienzyme extraction and high-performance liquid chromatography methods. *European Food Research and Technology* **2005,** *221* (1), 208-213.

58. De Quirós, A. R.-B.; De Ron, C. C.; Lopez-Hernandez, J.; Lage-Yusty, M., Determination of folates in seaweeds by high-performance liquid chromatography. *Journal of Chromatography A* **2004,** *1032* (1-2), 135-139.

59. Hoegger, D.; Morier, P.; Vollet, C.; Heini, D.; Reymond, F.; Rossier, J. S., Disposable microfluidic ELISA for the rapid determination of folic acid content in food products. *Analytical and bioanalytical chemistry* **2007,** *387* (1), 267-275.

60. Babakhanian, A.; Kaki, S.; Ahmadi, M.; Ehzari, H.; Pashabadi, A., Development of α-polyoxometalate–polypyrrole–Au nanoparticles modified sensor applied for detection of folic acid. *Biosensors and Bioelectronics* **2014,** *60*, 185-190.

61. Ensafi, A. A.; Karimi-Maleh, H., Modified multiwall carbon nanotubes paste electrode as a sensor for simultaneous determination of 6-thioguanine and folic acid using ferrocenedicarboxylic acid as a mediator. *Journal of Electroanalytical Chemistry* **2010,** *640* (1-2), 75-83.
62. Uysal, U. D.; Oncu-Kaya, E. M.; Tunçel, M., Determination of folic acid by CE in various cultivated variety of lentils. *Chromatographia* **2010,** *71* (7), 653-658.
63. Li, W.; Zhang, X.; Miao, C.; Li, R.; Ji, Y., Fluorescent paper–based sensor based on carbon dots for detection of folic acid. *Analytical and bioanalytical chemistry* **2020**, 1-9.
64. Sharma, A.; Arya, S., Economical and efficient electrochemical sensing of folic acid using a platinum electrode modified with hydrothermally synthesized Pd and Ag co-doped SnO2 nanoparticles. *Journal of The Electrochemical Society* **2019,** *166* (13), B1107.
65. Sharma, H. S.; Carmichael, E.; McCall, D., Fabrication of SERS substrate for the detection of rhodamine 6G, glyphosate, melamine and salicylic acid. *Vibrational Spectroscopy* **2016,** *83*, 159-169.
66. Li, Y.; Li, Q.; Wang, Y.; Oh, J.; Jin, S.; Park, Y.; Zhou, T.; Zhao, B.; Ruan, W.; Jung, Y. M., A reagent-assisted method in SERS detection of methyl salicylate. *Spectrochimica Acta Part A: Molecular and Biomolecular Spectroscopy* **2018,** *195*, 172-175.
67. Decorbie, N.; Tijunelyte, I.; Gam-Derouich, S.; Solard, J.; Lamouri, A.; Decorse, P.; Felidj, N.; Gauchotte-Lindsay, C.; Rinnert, E.; Mangeney, C., Sensing Polymer/Paracetamol Interaction with an Independent Component Analysis-Based SERS-MIP Nanosensor. *Plasmonics* **2020,** *15* (5), 1533-1539.
68. Yu, Q.; Zhao, Y.; Huang, L.; Sun, J.; Jin, D.; Shu, Y.; Xu, Q.; Hu, X.-Y., A flexible rGO electrode: a new platform for the direct voltammetric detection of salicylic acid. *Analytical Methods* **2020,** *12* (31), 3892-3900.
69. Chen, G.; Qiu, J.; Xu, J.; Wang, J.; Zhu, F.; Ouyang, G., Determination of four salicylic acids in aloe by in vivo solid phase microextraction coupling with liquid chromatography-photodiode array detection. *Talanta* **2018,** *184*, 520-526.
70. Ghoreishi, S. M.; Kashani, F. Z.; Khoobi, A.; Enhessari, M., Fabrication of a nickel titanate nanoceramic modified electrode for electrochemical studies and detection of salicylic acid. *Journal of Molecular Liquids* **2015,** *211*, 970-980.

71. Yuttakovit, S.; Santiwat, T.; Pratumyot, K.; Srikittiwanna, K.; Sukwattanasinitt, M.; Niamnont, N., A novel pyrenyl salicylic acid fluorophore for highly selective detection of paraquat in aqueous media. *Journal of Photochemistry and Photobiology A: Chemistry* **2020,** *397*, 112570.

72. Shahbazi, M.-A.; A Santos, H., Improving oral absorption via drug-loaded nanocarriers: absorption mechanisms, intestinal models and rational fabrication. *Current drug metabolism* **2013,** *14* (1), 28-56.
